# Examining driving stability and traffic capacity: A simulation study on appropriate speed limits in expressway work zones

**DOI:** 10.1371/journal.pone.0317690

**Published:** 2025-01-24

**Authors:** Qingwen Guo, Baohua Guo, Ziyan Zhao

**Affiliations:** 1 School of Energy Science and Engineering, Henan Polytechnic University, Jiaozuo, Henan, China; 2 Engineering Research Center of Road Transportation Decarbonization, Ministry of Education, Chang’an University, Xi’an, Shaanxi, China; 3 Jiaozuo Engineering Research Center of Road Traffic and Transportation, Henan Polytechnic University, Jiaozuo, Henan, China; Nanjing Forestry University, CHINA

## Abstract

In order to obtain proper speed limits in expressway work zones, CarSim and TruckSim software were used to determine the critical car and truck safe speeds and then VISSIM software was used to determine the traffic capacities and their corresponding speed limits under different upstream transition area lengths and road adhesion coefficients. The results show that critical car and truck safe speeds increase exponentially while traffic capacity and its corresponding speed limit increase logarithmically with rising road adhesion coefficient under a constant upstream transition area length, and critical car and truck safe speeds increase as a power function while traffic capacity and its corresponding speed limit increase exponentially with rising upstream transition area length under a constant road adhesion coefficient. Because Road Traffic Signs and Markings- Part 4: Work Zone (RTSM, GB 5768.4–2017) only gave the relationship between speed limit and upstream transition area length without considering road adhesion coefficient, the obtained determination method of optimal speed limit in expressway work zone in this paper has better reference value.

## 1. Introduction

With the rapid growth of traffic volume, some early built expressways in China that played a key role in the transportation system have entered the maintenance or major repair stage [1]. Therefore, they are often affected by expansion or upgrade measures, which are usually carried out while maintaining operations [2]. In expressway work zones, speed limits are usually much lower than normal operating speeds, and the reduction in the number of overtaking lanes caused by lane closures may create bottlenecks. Therefore, expressway work zones often become congestion points, which may reduce the traffic capacity of expressways and lead to traffic congestion [1]. Lochrane et al. [3] stated that work zones in particular cause about 24 percent of nonrecurring congestion and 10 percent of all congestion. In addition, forcing lane changes and merging in the work zone may lead to driver judgment errors and operational mistakes, resulting in traffic conflicts and endangering driving safety [4]. Accidents, their severity, and economic losses during expressway construction are notably higher than during normal road operations [5,6]. Hall and Lorenz’s [7] study revealed a 26% increase in accident rates during road maintenance, renovation, and expansion construction periods compared to non-construction periods. Zhao [8] observed a 2.7 times higher traffic accident rate in expressway work zones compared to non-construction road sections based on traffic accident data on expressways in Heilongjiang Province. According to the American Fatality Analysis Reporting System (FARS) [9], the number of traffic fatalities in road work zones accounts for about 3% of the total number of traffic fatalities, and rear end collisions account for 35% to 52% of these accidents. Speeding is the main cause of traffic accidents in road work zones [10].

**Fig 1:**
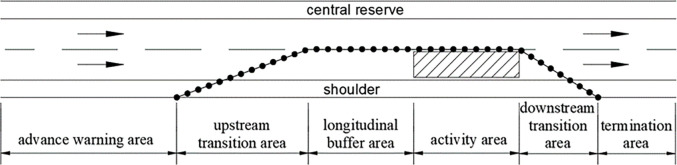
Layout plan of expressway work zones.

Understanding the impact of speed limits on the safety and efficiency of expressway work zones is crucial for enhancing driving stability and traffic capacity. Currently, traffic organization schemes for expressway work zones in China primarily adhere to standards such as the Technical Specification of Maintenance for Highway (TSMH, JTG H10-2009 [11]), RTSM (Road Traffic Signs and Markings- Part 4: Work Zone, GB5768.4–2017 [12]), and Safety Work Rules for Highway Maintenance (SWRHM, TG H30-2015 [13]), along with management measures developed by public authorities. These standards and methods offer broad regulations and guidance for traffic safety management and control facilities at work zones. For instance, the SWRHM outlines the components of expressway work zone, including advance warning area, upstream transition area, longitudinal buffer area, activity area, downstream transition area, and termination area (Fig 1).

Domestic and foreign scholars have conducted extensive research on speed limit issues in expressway work zones. Dudek and Richard [14] pointed out that setting speed limits can improve road traffic safety. Lu [15] suggested incorporating traffic volume into the calculation of speed limits in the work zones. Yu [16] proposed a method of weighting safety and efficiency characteristic indicators to determine the speed limit value of the work zone. In terms of the impact of road adhesion coefficient on speed limit, Guo et al. [17] analyzed the influence of adverse weather conditions on traffic capacity, determined the maximum speed limit value of roads under adverse weather conditions, and emphasized the importance of road adhesion coefficient in speed limit decision-making. Shin et al. [18] used TruckSim simulation software to analyze the driving behavior of vehicles, indicating that sideslip is mainly influenced by the road adhesion coefficient. Wang et al. [19] used CarSim software to simulate vehicle driving scenarios under different road adhesion coefficients. Research has shown that road adhesion coefficients between 0.10 and 0.20 may cause sideslip, while road adhesion coefficients between 0.21 and 0.35 may directly lead to vehicle rollover. The above research indicates that the setting of speed limit values should consider the impact of road adhesion coefficient on vehicle stability.

In terms of speed limit schemes for expressway work zones, Yu et al. [20] studied various speed limit schemes for two-way four lane expressway work zones and found that the conflict rate in the upstream transition area was significantly higher than that in the advance warning area, emphasizing that the length of the upstream transition area has a significant impact on traffic efficiency and safety. Ding et al. [21] obtained the model of the influence of the length and speed limit of the upstream transition area on traffic safety and vehicle mobility through regression analysis. The research results provide scientific basis for the optimization of the length and speed limit of the upstream transition area setting in work zones. Ma et al. [22] analyzed traffic organization schemes for work zones with different speed limits and upstream transition area lengths to find the optimal operational quality and service level for expressway work zones under specific conditions. Yu et al. [23] found that implementing phased speed limit procedures for vehicles entering expressway work zones improved the overall safety of vehicle driving and construction operations.

In addition, researchers also used various simulation methods to simulate the proper speed limit in the work zone. Liang et al. [24] conducted driving simulation tests in expressway work zones. Wu et al. [25] used VISSIM software to check vehicle operations in the expressway work zones under various speed limits. Liu et al. [26] combined driving simulation with VISSIM to verify the rationality of the speed limit results from the perspectives of driver adaptability and traffic flow operation status.

In the research of traffic conflict evaluation in the speed limit scheme of the work zones, Gossai et al. [27] used the Surrogate Safety Assessment Model (SSAM) and VISSIM to verify the effectiveness of speed limits in improving safety by comparing the number of traffic conflicts under different speed limit strategies. Mohamed et al. [28] used VISSIM to test speed limit strategies and obtained collision conflicts through SSAM to evaluate the impact of different speed limit strategies on improving weaving area safety. Ge et al. [29] used VISSIM software and SSAM to determine advance warning zone length of expressway, and studied the relationship between advance warning zone length and safety evaluation indicators.

**Fig 2:**
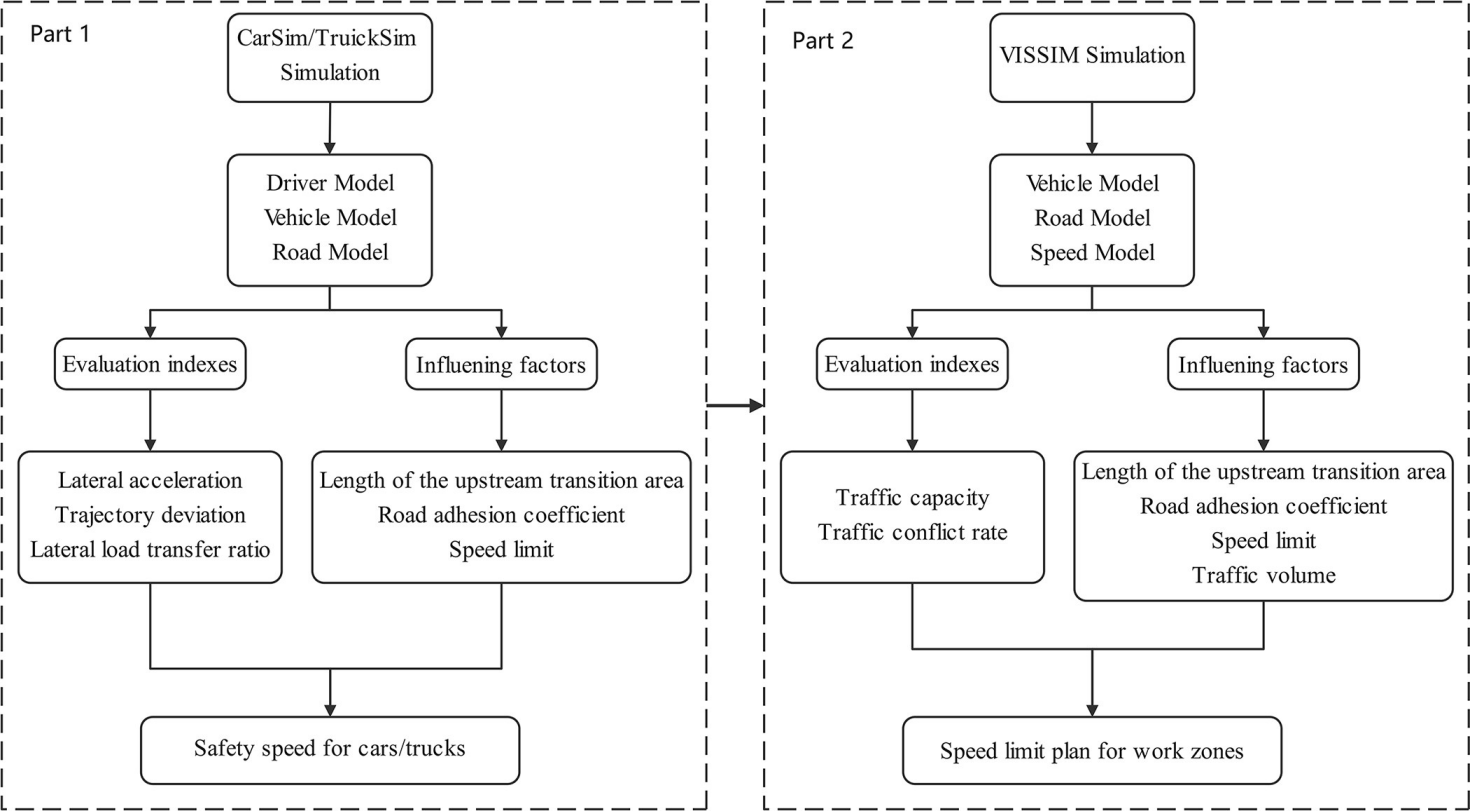
Simulation flow chart.

In summary, some researches have been conducted on the impact of road adhesion coefficient on driving stability, the relationship between speed limits in expressway work zones and the length of upstream transition areas, and the influence of traffic conflict rate on speed limit strategies. However, there is a lack of exploration on the impact of road adhesion coefficient and traffic capacity in the study of the relationship between speed limit strategies in expressway work zones and the length of upstream transition areas. This paper intends to first determine the critical safe speed under different upstream transition area lengths and road adhesion coefficients based on the driving stability of vehicles. Then, within the range below the critical safe speed, the traffic capacity and corresponding speed limit of the expressway work zone will be studied in relation to the length of the upstream transition area and road adhesion coefficient, in order to determine the optimal speed limit strategy for the expressway work zone. The main organization of this paper is as follows, Section 2 and Section 3 introduce the simulation method and analyze the simulation results of CarSim, TruckSim and VISSIM software respectively. The detailed settings of the simulation process are shown in the Fig 2. Section 4 and Section 5 are respectively the discussion and conclusion.

## 2. Determination of critical safe speed in work zone based on driving stability

### 2.1 Simulation model

**Fig 3:**
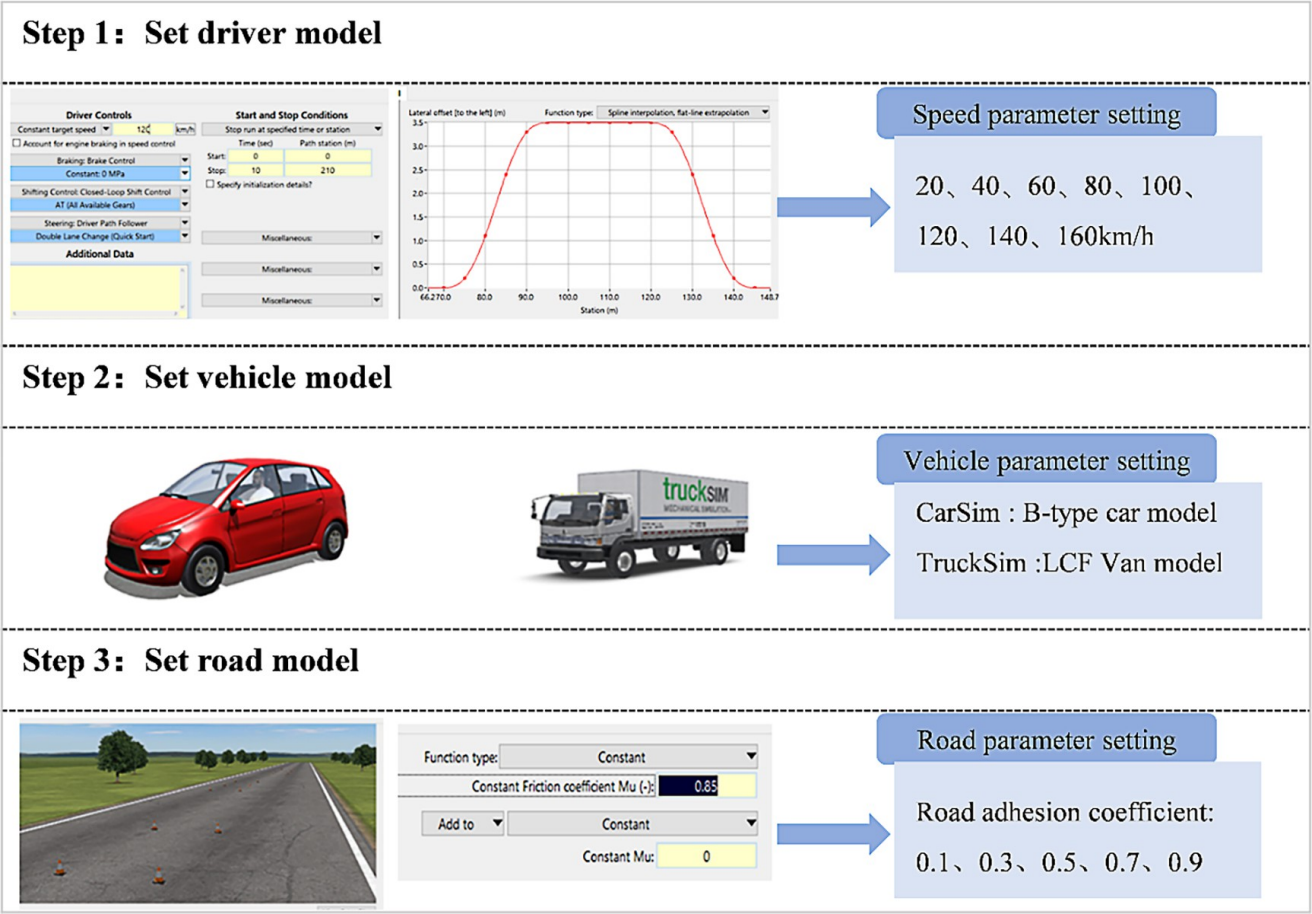
Simulation process and parameter settings in CarSim and Trucksim.

CarSim and TruckSim software can be used to simulate vehicle dynamic behavior, including handling, braking, power, and response to road and driver inputs [30]. This study used CarSim and TruckSim to analyze the effects of the length of the upstream transition area, road adhesion coefficient, and driving speed on the stability of cars or trucks in the expressway work zone. The purpose is to establish the relationship between the critical safe speed in the expressway work zone, the length of the upstream transition area, and the road adhesion coefficient. The specific model establishment process is shown in Fig 3.

The driver model is responsible for controlling the speed and direction of the vehicle during the vehicle dynamics simulation process. The vehicle speed value is set to a fixed value of 20 to 160 km/h, and the vehicle follows a predetermined sine curve trajectory within the upstream transition area [31]. The length value of the upstream transition area is set to a value of 20 to 100 m, while other parameters remain at default values in the software. CarSim simulation uses the B-type car model, while TruckSim simulation uses the LCF Van model. The default parameters in CarSim/TruckSim software are selected as the vehicle model parameters for their respective simulations. Configure a two-way four lane road model by entering road parameters in the "Additional Data" section of CarSim/TruckSim software. The lane width is set to 3.75m, and the road adhesion coefficient ranges from 0.1 to 0.9. Other road model parameters are kept at default values in the software.

### 2.2 Evaluation indexes

The evaluation indexes include sideslip index, trajectory deviation index, and rollover index. The sideslip index is the lateral acceleration of the vehicle. According to the national standard [32], the critical value for trucks is 0.3g, and for sedans it is 0.4g. Here, ’g’ represents the gravitational acceleration. The trajectory deviation index evaluates the lateral control stability of a vehicle based on the trajectory deviation distance in the longitudinal distance trajectory deviation curve. According to the "comprehensive variance evaluation method", when the trajectory deviation measurement value is less than 0.3 meters [33], it indicates the close alignment between the actual driving trajectory and the target trajectory line. During the driving process of the vehicle, the force on the wheels perpendicular to the ground will vary with the tilt angle of the vehicle body. The rollover evaluation index adopts the lateral load transfer ratio (LTR), which is an index for evaluating the instability of vehicle rollover. The expression for LTR is as follows:

$$LTR=\left|\frac{\sum_{i=1}^{n}\left(F_{li}-F_{ri}\right)}{\sum_{i=1}^{n}\left(F_{li}+F_{ri}\right)}\right|,$$ (1)

where *F*_*li*_ is the vertical load on the left wheel of the vehicle; *F*_*ri*_ is the vertical load on the right wheel of the vehicle; *i* is the axle number; *n* is the total number of axles. In this paper, the vehicle speed at LTR = 0.6 is considered as the critical safe speed for vehicle rollover [34].

### 2.3 Simulation results

#### 2.3.1 CarSim simulation results

**Fig 4:**
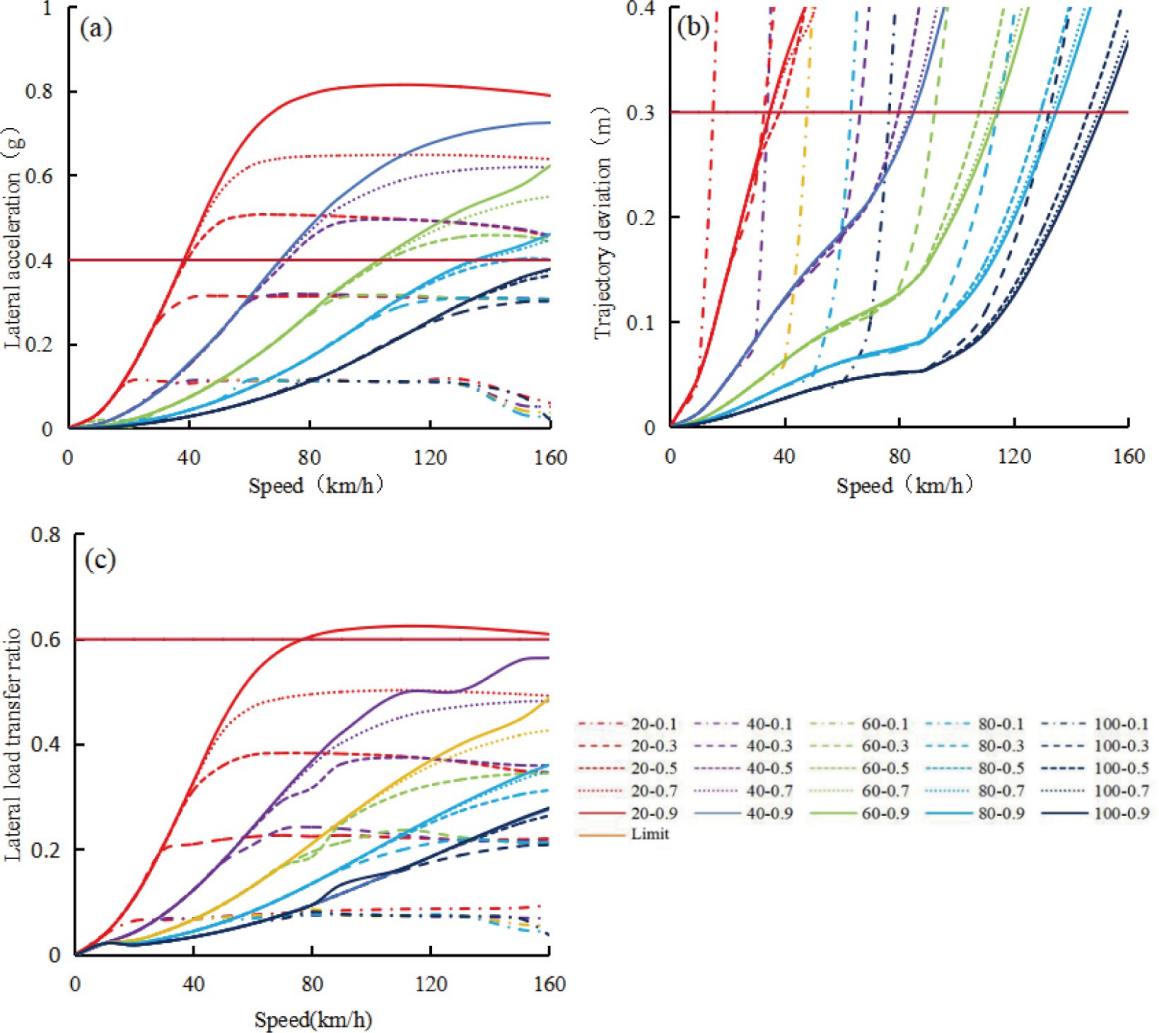
Relationship between evaluation indexes and influencing factors for car. (a) Lateral acceleration; (b) Trajectory deviation value; (c) Lateral load transfer ratio.

Using CarSim software simulation, the relationship between evaluation indexes including lateral acceleration, trajectory deviation value, lateral load transfer ratio, and influencing factors such as vehicle speed, upstream transition area length, and road adhesion coefficient was established, as shown in Fig 4. In the legend of Fig 4, taking 20–0.1 as an example, it indicates that the length of the upstream transition area is 20m and the road adhesion coefficient is 0.1.

In Fig 4A, the lateral acceleration of the vehicle shows four stages: acceleration increase, uniform increase, deceleration increase, and stabilization with changes in vehicle speed, while maintaining a constant length of the upstream transition area and road adhesion coefficient. When the length of the upstream transition area is fixed, regardless of the road adhesion coefficient, the lateral acceleration remains the same during the acceleration and uniform increase stages. During the deceleration and stabilization phases, both the lateral acceleration and the speed entering the stabilization phase increase with the increase of road adhesion coefficient. Similarly, under a constant road adhesion coefficient, the lateral acceleration decreases as the length of the upstream transition area increases, and the speed increases when entering the stable phase. The speed corresponding to the critical lateral acceleration of 0.4g also increases with the increase of the length of the upstream transition area and the decrease of the road adhesion coefficient.

In Fig 4B, as the vehicle speed increases, the trajectory deviation of the car shows two longer approximately uniform increasing stages, with a shorter acceleration increasing stage in between, while the length of the upstream transition area and the road adhesion coefficient remain unchanged. When the road adhesion coefficient remains constant, the trajectory deviation decreases as the length of the upstream transition area increases. Similarly, with a constant length of the upstream transition area, trajectory deviation decreases as the road adhesion coefficient increases. Under each length of upstream transition area and road adhesion coefficient, all maximum trajectory deviation values exceed 0.3 m in the range of speeds below 160 km/h. As the length of the upstream transition area and the road adhesion coefficient increase, the corresponding speed will also increase when the trajectory deviation exceeds 0.3m.

In Fig 4C, the variation of the lateral load transfer ratio of the vehicle with speed is similar to the lateral acceleration of the vehicle in Fig 4A under different upstream transition area lengths and road adhesion coefficients. When the length of the upstream transition area and the road adhesion coefficient remain constant, the lateral load transfer ratio increases with the increase of speed, showing four stages: acceleration increase, uniform increase, deceleration increase, and basic stability. The influence of road adhesion coefficient on the lateral load transfer ratio is relatively small in the acceleration and uniform increase stages, but relatively large in the deceleration and basic stability stages. Increasing the length of the upstream transition area will reduce the lateral load transfer ratio at different stages at the same speed, while keeping the road adhesion coefficient constant. Only when the length of the upstream transition area is 20 m and the road adhesion coefficient is 0.9, can the critical value of the lateral load transfer ratio of 0.6 be reached, corresponding to a critical safe speed of about 75 km/h.

**Fig 5:**
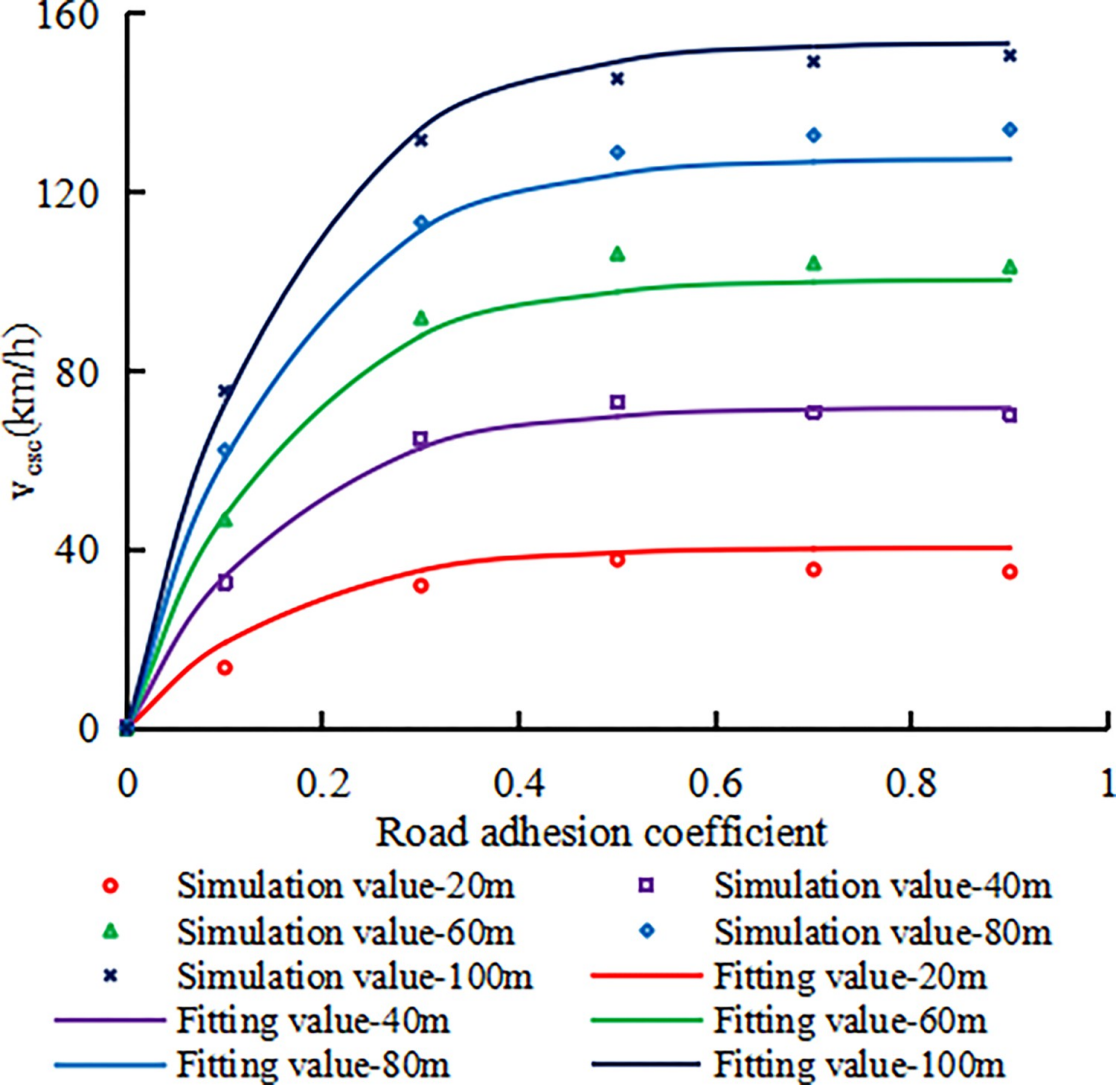
The relationship between the critical safe speed and the length of the upstream transition area and the road adhesion coefficient for cars.

Under different upstream transition area lengths and road adhesion coefficients, when the fitted curve in Fig 4 intersects with a lateral acceleration of 0.4g, a trajectory deviation of 0.3m, and a lateral load transfer ratio of 0.6, the speed corresponding to this intersection point is a candidate critical safe speed value. Under the same length of the upstream transition area and road adhesion coefficient, the minimum value among these three candidate critical safe speeds is determined as the critical safe speed value of vehicle. Fig 5 shows the relationship between the critical safe speed value of a vehicle and the length of the upstream transition area and the road adhesion coefficient. When the road adhesion coefficient or the length of the upstream transition area is zero, the theoretical critical safe speed is also zero, as shown in Fig 5. From Fig 5, it can be seen that at each upstream transition area length, the critical safe speed of the vehicle first decelerated increases with the increase of road adhesion coefficient, and then reaches a platform. However, for a constant road adhesion coefficient, the critical safe speed of the vehicle increases approximately uniformly with the increase of upstream transition area length. The relationship between the critical safe speed for a car (v_csc_), upstream transition area length (L) and road adhesion coefficient (μ) can be expressed by regression analysis as follows:

$$v_{csc}=3.36L^{0.83}(1-exp(-7.52\mu^{1.07}))(R^{2}=0.9964).$$ (2)

From Formula (2), it can be seen that when the road adhesion coefficient remains constant, the critical safe speed of the vehicle has a power function relationship with the length of the upstream transition area. As the length of the upstream transition area increases, the critical safe speed of the vehicle decelerated increases. When the length of the upstream transition area remains constant, there is a power function relationship between the critical safe speed of the vehicle and the road adhesion coefficient. As the road adhesion coefficient increases, the critical safe speed of the vehicle also increases, consistent with the previous analysis.

#### 2.3.2 TruckSim simulation results

**Fig 6:**
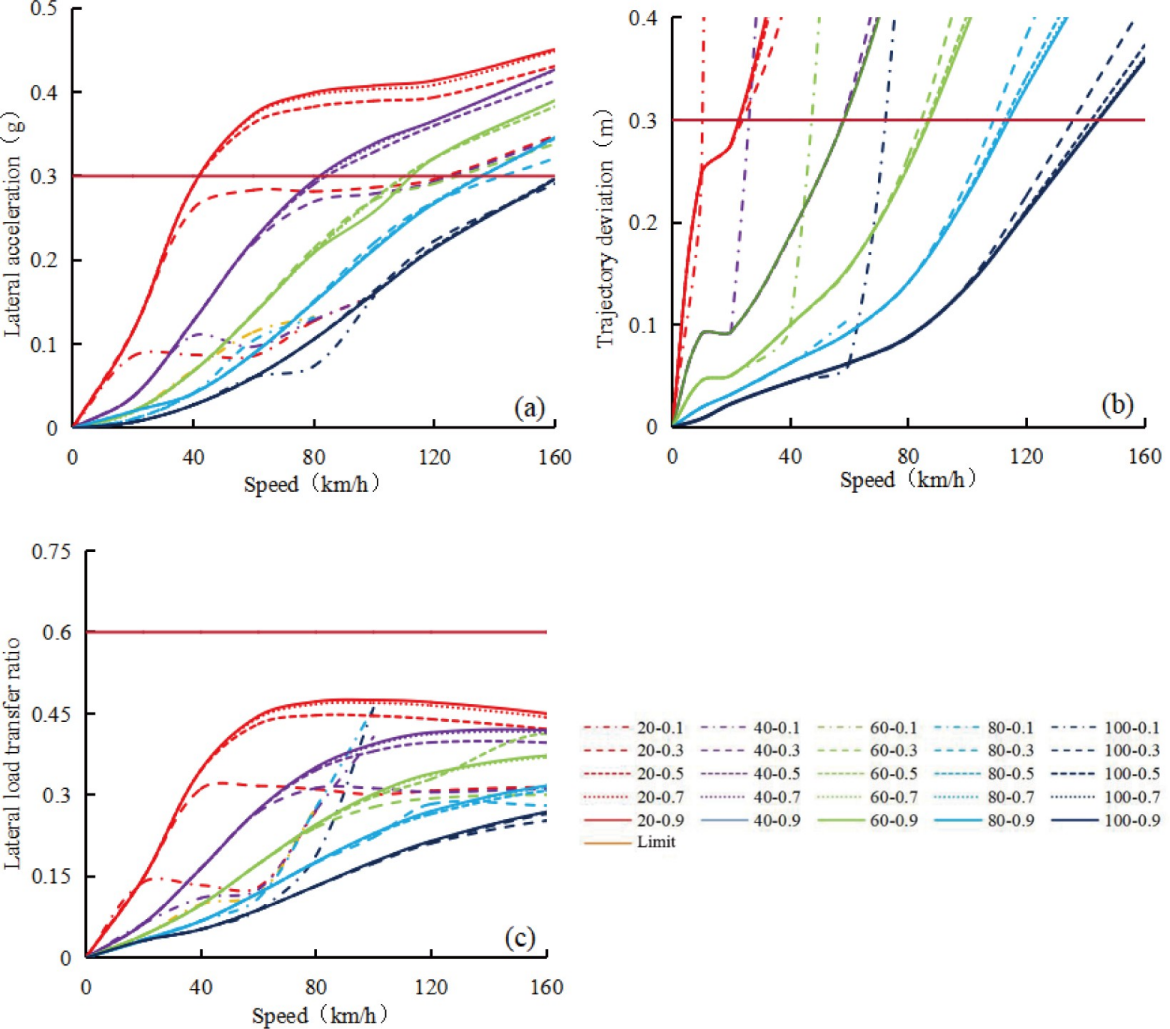
Relationship between evaluation indexes and influencing factors for truck. (a) Lateral acceleration; (b) Trajectory deviation value; (c) Lateral load transfer ratio.

The simulation results of TruckSim software reveal the relationship between evaluation indexes including lateral acceleration, trajectory deviation value, lateral load transfer ratio, and influencing factors including vehicle speed, upstream transition area length, and road adhesion coefficient, as shown in Fig 6. Fig 6 shows that the relationship between the lateral acceleration, trajectory deviation, and lateral load transfer ratio of the truck and the truck speed at various upstream transition area lengths and road adhesion coefficients is similar to the results obtained from car simulation using CarSim software. Therefore, further explanation of these similarities is omitted here. However, the platform stage of the truck’s lateral acceleration in Fig 6A is not obvious in a large speed range. When the speed is 160km/h, the lateral acceleration of the truck still shows an upward trend with increasing speed, which is not completely consistent with the performance of the car in Fig 5A.

**Fig 7:**
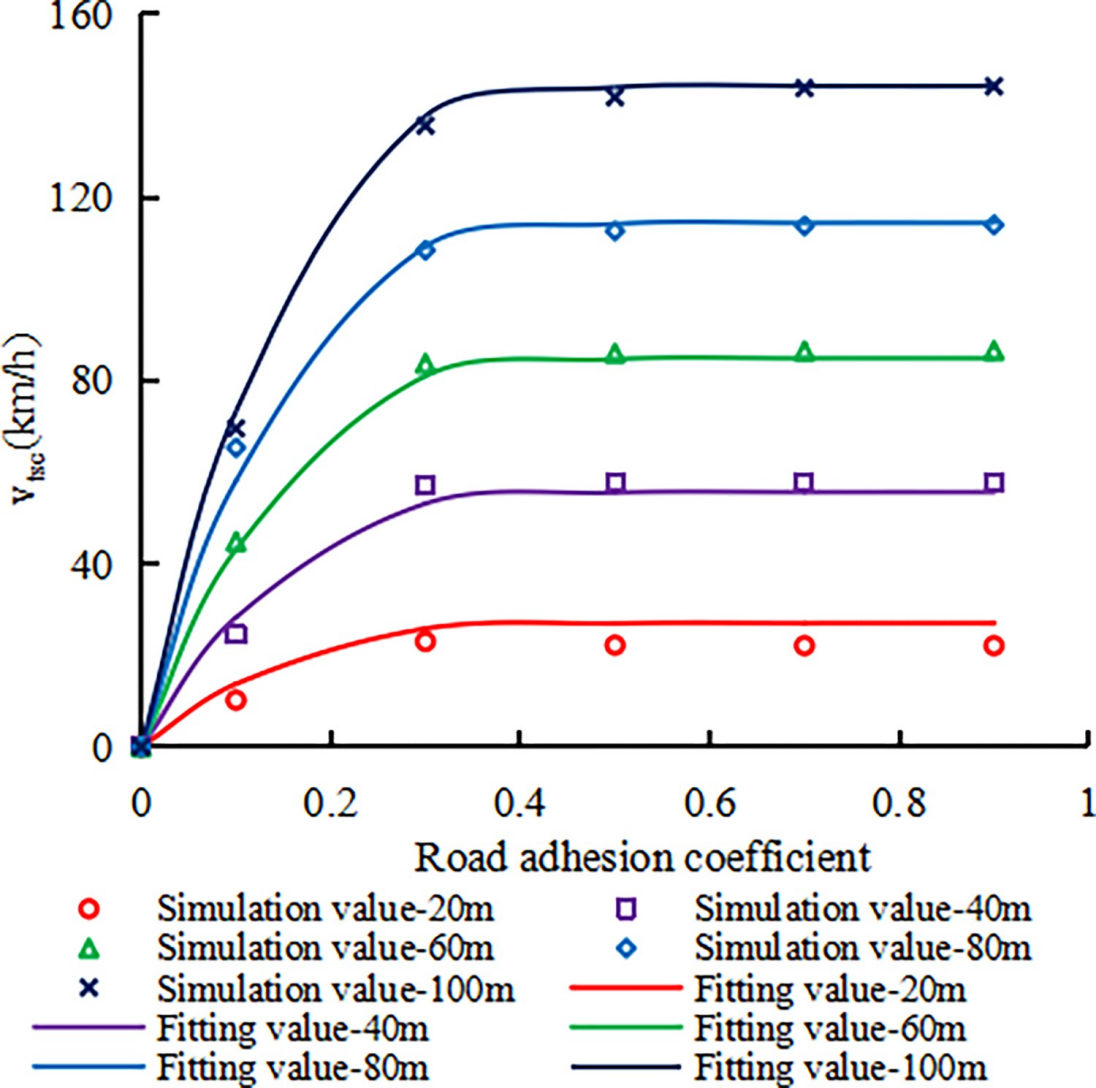
The relationship between the critical safe speed and the length of the upstream transition area and the road adhesion coefficient for trucks.

Similarly, for each upstream transition area length and road adhesion coefficient, three candidate critical safe speed values are determined based on the intersection points of the fitting curve of the simulation results and the lateral acceleration of 0.3g, trajectory deviation of 0.3m, and lateral load transfer ratio of 0.6. The minimum critical safe speed value of the truck is determined as the critical safe speed value of the truck. Fig 7 shows the relationship between the critical safe speed value of the truck and the length of the upstream transition area and the road adhesion coefficient. In theory, when the road adhesion coefficient is 0 or the length of the upstream transition area is 0m, the critical safe speed of the truck is 0km/h, as shown in Fig 7.

From Fig 7, it can be seen that at each upstream transition area length, the critical safe speed of the truck decelerated increases to a basically constant stage as the road adhesion coefficient increases. When the road adhesion coefficient is constant, the critical safe speed of the truck increases approximately uniformly with the length of the upstream transition area. The relationship between the critical safe speed of the truck (v_tsc_) and the length (L) of the upstream transition area and the road adhesion coefficient (μ) was determined through regression analysis as Formula (3), which is completely identical in form to Formula (2).


$$v_{tsc}=1.2L^{1.04}(1-exp(-15.52\mu^{1.34}))(R^{2}=0.9974).$$ (3)

## 3. Determination of speed limit in expressway work zone based on traffic capacity

### 3.1 Model establishment

**Fig 8:**
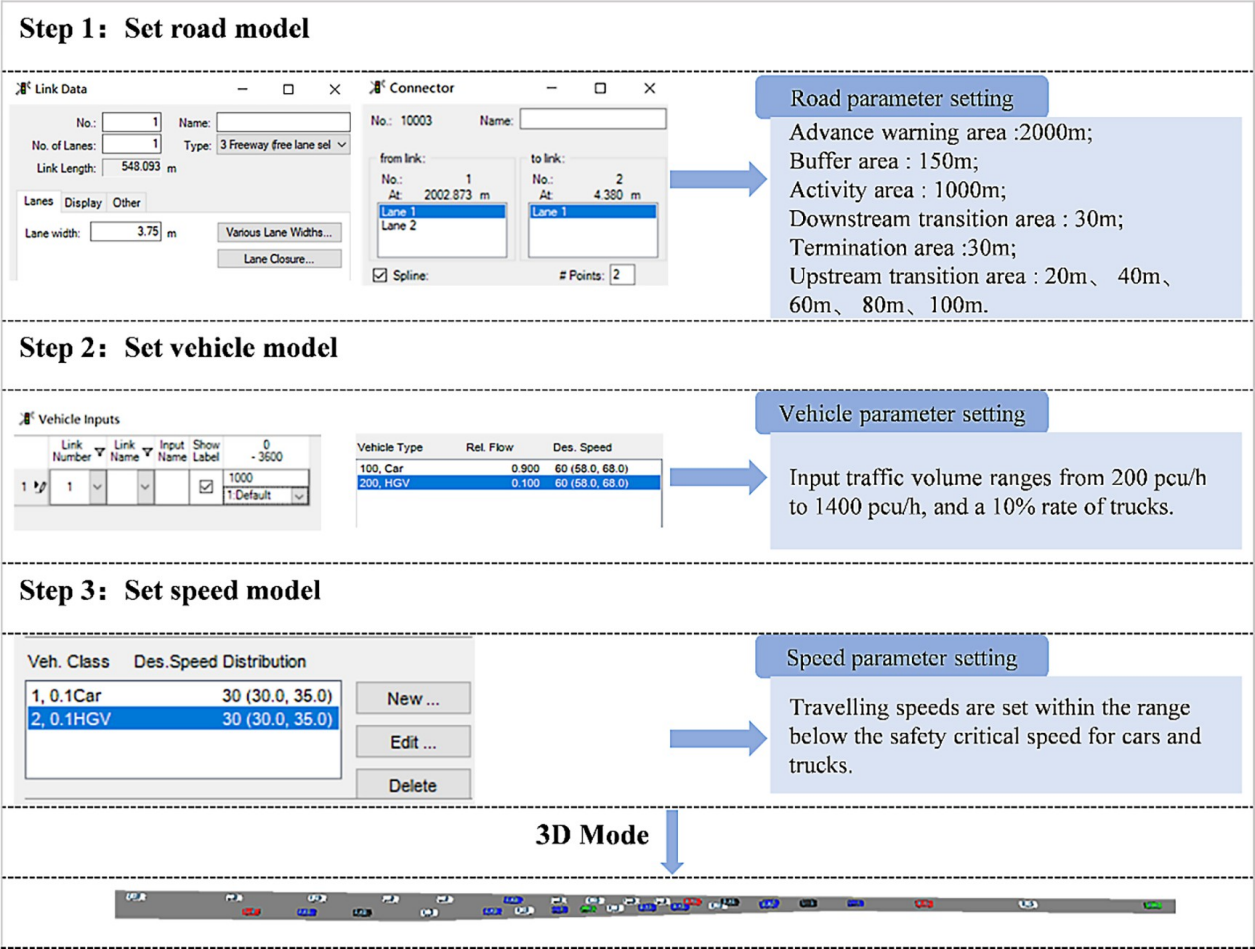
Simulation process and parameter setting in VISSIM.

VISSIM software is developed by the German company PTV and is a discrete, random, and microscopic traffic flow simulation system. It is widely used to simulate and analyze urban and public transportation operations under different traffic conditions, relying on time intervals and driving behavior. After using CarSim and TruckSim software to determine the critical safe speed values for cars and trucks in the expressway work zone, VISSIM simulation software was used to study the relationship between the maximum traffic capacity and its corresponding speed limit value in the expressway work zone, the length of the upstream transition zone, and the road adhesion coefficient within the critical safe speed range. The specific model establishment process is shown in Fig 8.

The road segment model parameters used in VISSIM software are consistent with those used in CarSim and TruckSim software, and also use a two-way four lane road model with a lane width set to 3.75m. The expressway work zone model includes a 2000 m long advance warning area, a 150m long buffer area, a 1000m long activity area, and a 30m long downstream transition area and termination area. The length of the upstream transition area is set as a variable in the simulation, with values of 20m, 40m, 60m, 80m, and 100m, respectively. The range of road adhesion coefficient is 0.1 to 0.9, with a set interval of 0.2. The model adopts the gradual speed limit scheme of SWRHM, starting from the starting point of the advance warning area, reducing the vehicle speed by 20 km/h or 10 km/h every 200m or 100m until it reaches the designated speed limit at the starting point of the upstream transition area.

**Fig 9:**
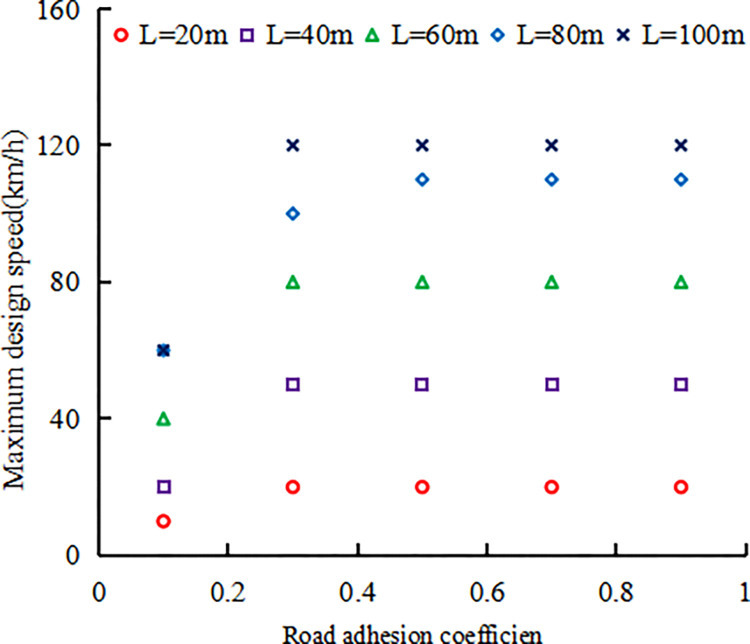
Maximum design speed.

The traffic conditions in VISSIM simulation are set as follows: the input traffic volume ranges from 200pcu/h to 1400pcu/h, with intervals of 200pcu/h, and a truck blending rate of 10%. Under each upstream transition area length and road adhesion coefficient, the speed limit is set below the critical safe speed for the car and truck. The speed limit is set from 10km/h, with each scheme increasing by 10km/h until the critical safe speed determined in Figs 5 and 7. Considering that the maximum speed limit on Chinese expressways is 120 km/h, when the critical safe speed in Figs 5 and 7 exceeds 120 km/h, the maximum speed limit in VISSIM simulation is set to 120 km/h. The maximum speed limit designed for different upstream transition area lengths and road adhesion coefficients is shown in Fig 9.

According to the theory of vehicle dynamics [35], the expression for maximum acceleration is as follows:

$$a_{p}=k_{1}\mu g,$$ (4)

$$k_{1}=\frac{\frac{l_{v}}{l_{a}}}{1-\frac{h}{l_{a}}},$$ (5)

where *a*_*p*_ is the maximum acceleration, m/s^2^; *μ* is the road adhesion coefficient; g is the gravitational acceleration, m/s^2^; *l*_*v*_ is the distance from the mass centre to the front axle, m; *l*_*a*_ is the axle distance, m; *h* is the height of mass centre, m; *k*_1_ is the influence coefficient of gravity centre position, which is determined by the default maximum car acceleration of 3.5 m/s^2^ and the default maximum truck acceleration of 7.3 m/s^2^ in VISSIM, and the normal road adhesion coefficient of 0.75, and the calculated *k*_1_ are 0.47 for cars and 0.97 for trucks, respectively.

The expression of maximum deceleration *a*_*m*_ is as follows:

$$a_{m}=k_{2}\mu g,$$ (6)

where *a*_*m*_ is the maximum deceleration, m/s^2^; *k*_2_ is the ratio of the truck’s maximum deceleration to the car’s maximum deceleration, which is 0.73 by VISSIM default.

The Wiedemann model, tailored for expressway, is chosen here. The minimum stopping spacing in the model is adjusted based on road adhesion coefficient for each vehicle speed using the following formula [36,37]:

$$l_{0}=l_{1}+l_{2}+l_{3}+l_{4}=\frac{vt}{3.6}+\frac{v^{2}}{254\mu }+l_{3}+l_{4},$$ (7)

where *l*_0_ is minimum headway, m; *t* is driver’s perceptual reaction time, 2.5s; *v* is vehicle speed, km/h; *l*_1_ is the driving distance in the driver’s reaction time, m; *l*_2_ is vehicle braking distance, m; *l*_3_ is minimum safety spacing between adjacent vehicles, m, 2 m; *l*_4_ is vehicle length, m, 5m; *μ* is road adhesion coefficient.

After establishing the simulation model and setting the parameters, the simulation process can begin. Each simulation scheme runs for 3600 seconds, and the parameter values of each simulation scheme are taken as the average of 5 simulation results.

### 3.2 Evaluation indexes

By using traffic capacity as an efficiency index and traffic conflict rate as a safety index, the optimal speed limit for the expressway work zone is established to balance safety and efficiency [38,39]. To prevent tailgate conflicts between vehicles, Formula (7) was used in the simulation to establish the minimum headway. Subsequently, according to Formula (8), the theoretical traffic capacity of the lane *q*_*c*_ is determined as follows:

$$q_{c}=\frac{1000v}{l_{0}}.$$ (8)

Formula (8) indicates that when the minimum headway is constant, the theoretical traffic capacity of the road increases with the increase of vehicle speed. However, according to Formula (7), the minimum headway will increase with the increase of vehicle speed, and the theoretical traffic capacity of the road may also decrease with the increase of vehicle speed. Therefore, the impact of increasing vehicle speed on the theoretical traffic capacity of roads is still uncertain. The VISSIM software can be used to study the relationship between theoretical road traffic capacity and speed limits. The acquisition of parameters such as vehicle speed, traffic flow, and conflict rate can be obtained by configuring them in the VISSIM software.

The traffic conflict rate is the ratio of the number of traffic conflicts caused by vehicles passing through the road work zone per unit time to the length of the road working area and the number of vehicles passing through. Its formula is as follows:

$$f=\frac{F_{TC}}{ql},$$ (9)

where *f* represents the traffic conflict rate in the work zone, measured in freq/(pcu·km), it is determined by the number of traffic conflicts caused by vehicles passing through a unit of times *F*_*TC*_, the hourly traffic volume *q* within the work zone (pcu), and the length *l* of the work zone (km).

SSAM was developed by the Federal Highway Administration (FHWA) in the United States [40]. It uses analysis indexes such as TTC (Time To Collision) and PET (Time After Invasion), and uses the “Vehicle Tracking Record File” generated by VISSIM software to obtain the number of traffic conflicts per unit hour *F*_*TC*_. Specifically, *F*_*TC*_ can be obtained by importing the “Vehicle Tracking Record File” into SSAM software, and then using Formula (9) to calculate the traffic conflict rate *f*.

### 3.3 VISSIM simulation results

#### 3.3.1 Relationship between traffic capacity and input traffic volume

**Fig 10:**
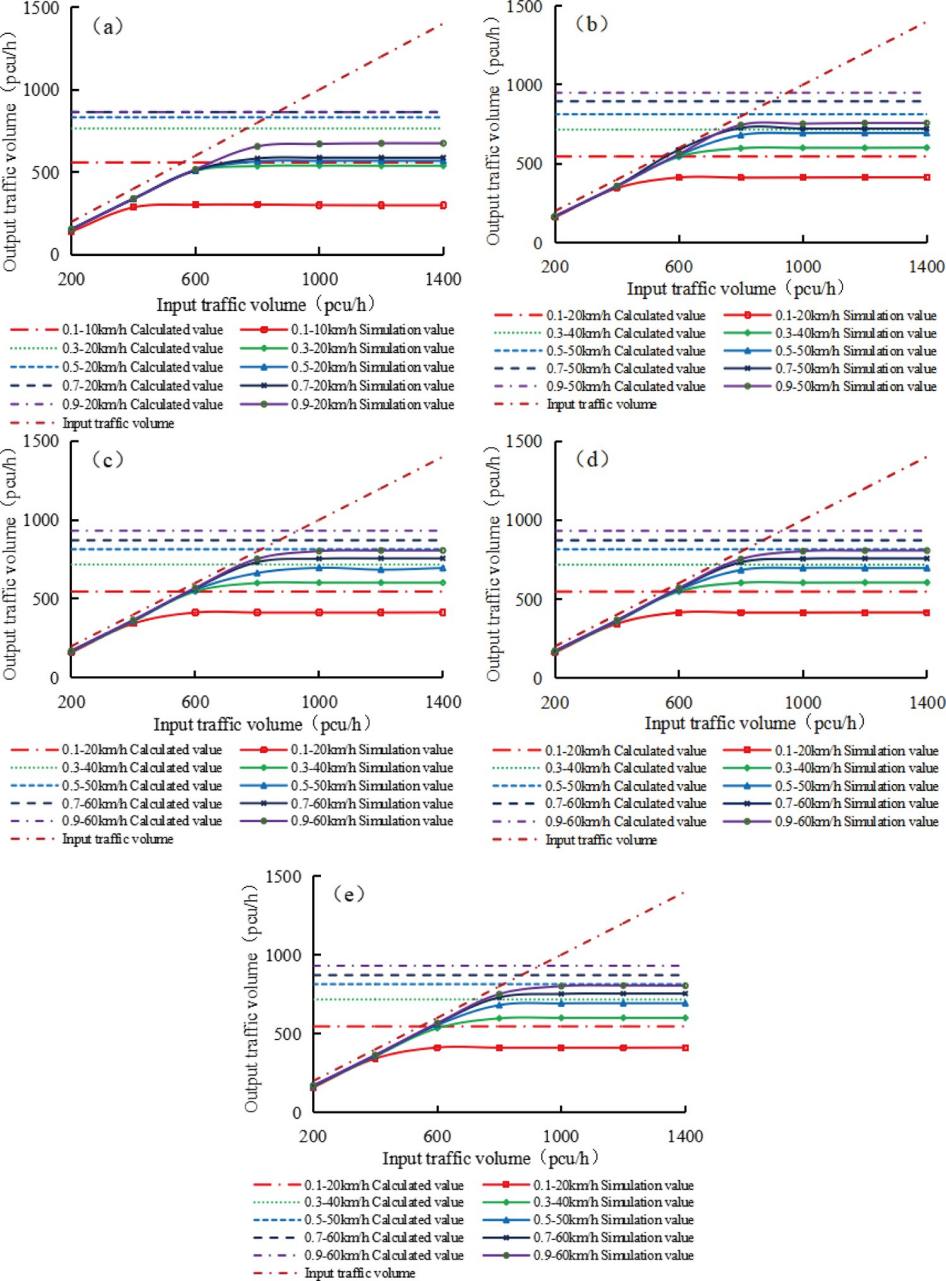
Relationship between the simulated and calculated traffic capacity and input traffic volume.

Based on the maximum speed values of various upstream transition area lengths and road adhesion coefficients given in Fig 9, different speed limit schemes were set, and seven different input traffic volumes were used for each speed limit scheme. VISSIM software was used to simulate and study the traffic operation status of the expressway work zone under the above schemes. Under different speed limit schemes, the simulated traffic capacity varies depending on the length of the upstream transition area and the road adhesion coefficient. Here, the focus is on the maximum traffic capacity and its corresponding speed limit, the simulated and calculated maximum traffic capacities within the speed limit scheme for different lengths of upstream transition zones and road adhesion coefficients are shown in Fig 10.

(a) L = 20m; (b) L = 40m; (c) L = 60m; (d) L = 80m; (e) L = 100m.

In Fig 10, under each upstream transition area, the calculated traffic capacity increases with the increase of adhesion coefficient, but does not change with the increase of input traffic volume; the simulated traffic capacity increases with the increase of road adhesion coefficient, and the growth rate becomes smaller and smaller; as the input traffic volume increases, the simulated traffic capacity first increases uniformly and then enters a platform, which basically does not change with the increase of input traffic volume. At each road adhesion coefficient, the calculated traffic capacity is a horizontal straight line that intersects with the input traffic volume straight line. Before and after the intersection, the calculated traffic capacity changes from greater than the input traffic volume to less than the input traffic volume, and the input traffic volume corresponding to the intersection increases with the increase of road adhesion coefficient. Under different road adhesion coefficients, the calculated traffic capacity and input traffic volume are both greater than the simulated traffic capacity under the same road adhesion coefficient.

Fig 10 also shows that after the input traffic volume exceeds the calculated traffic capacity, the simulated traffic capacity tends to stabilize and no longer changes significantly with the increase of input traffic volume. As the road adhesion coefficient increases, the simulated traffic capacity when entering the platform phase will also increase. But in all cases, when the input traffic volume is 1000pcu/h, regardless of the length of the upstream transition area or the road adhesion coefficient, the simulated traffic capacity enters the platform stage. Therefore, in all simulation schemes, the input traffic volume is set to 1000pcu/h, which can prevent insufficient input traffic volume and prevent excessive traffic volume with invalid input.

#### 3.3.2 Optimal speed limit based on traffic capacity

**Fig 11:**
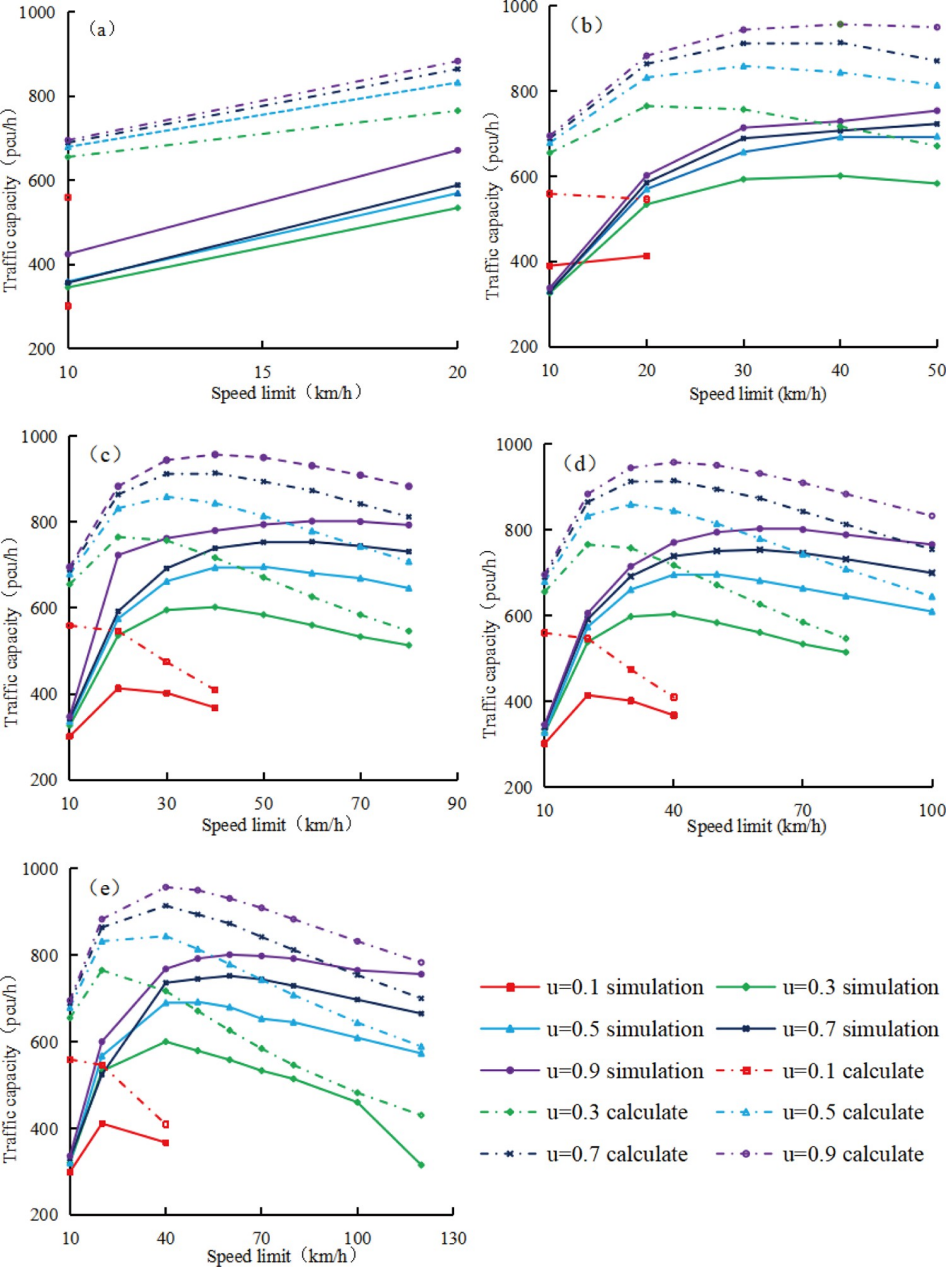
Relationship between the calculated and simulated traffic capacities and the speed limit in the work zone. (a) L = 20m; (b) L = 40m (c) L = 60m; (d) L = 80m; (e) L = 100m.

Fig 11 shows the correlation between calculated and simulated traffic capacities and speed limit values under different upstream transition area lengths and road adhesion coefficients with an input traffic volume of 1000 pcu/h.

In Fig 11, Calculated traffic capacity shows decreasing trend at road adhesion coefficient of 0.1. When the length of the upstream transition area is 20m, the calculated and simulated traffic capacities show a upward trend with the increase of speed limit. In other cases, the calculated and simulated traffic capacities first increase and then decrease with the increase of speed limit. Overall, under various speed limits, the calculated traffic capacity always exceeds the simulated value, but as the vehicle speed limit increases, this difference between the calculated and simulated traffic capacities will decrease. Additionally, under constant upstream transition area length and vehicle speed limit, both the calculated and simulated traffic capacities rise with the road adhesion coefficient. Yet, the difference between the calculated and simulated traffic capacities decrease as the road adhesion coefficient increases.

**Fig 12:**
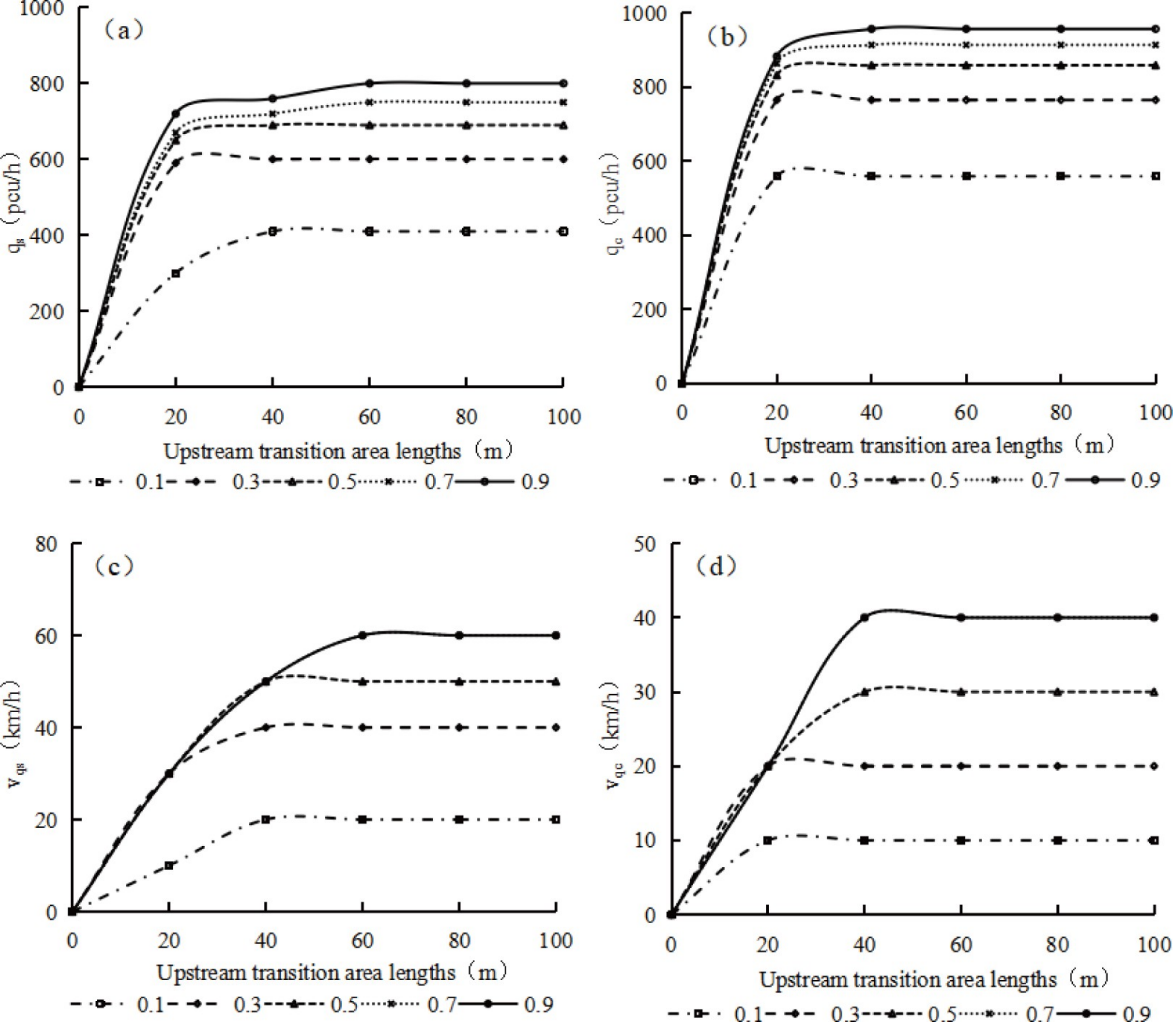
The calculated and simulated traffic capacities and their corresponding vehicle speed limits under different upstream transition area length and road adhesion coefficient. (a) *q*_*s*_ (b) *q*_c_ (c) *v*_qs_ (d) *v*_qc_.

The calculated traffic capacity *q*_c_ and simulated traffic capacity *q*_s_ and their corresponding speed limits *v*_qc_ and *v*_qs_ under each upstream transition area length and road adhesion coefficient can be obtained as shown in Fig 12. When the upstream transition area length is 0 m or the road adhesion coefficient is 0, *q*_s_ is 0 pcu/h and its corresponding speed is also 0 km/h. From Fig 12A and 12B, it can be seen that when the road adhesion coefficient is certain, both *q*_s_ and *q*_c_ decelerated increase with the increase of upstream transition area length until they enter a constant value. When the upstream transition area length is certain, both *q*_s_ and *q*_c_ decelerated increase with the increase of road adhesion coefficient.

From Fig 12C and 12D, it can be seen that when the road adhesion coefficient is constant, the vehicle speeds corresponding to the simulated and calculated traffic capacities decelerated increases with the length of the upstream transition area until constant values are reached. When the length of the upstream transition area is constant, except for a road adhesion coefficient of 0.9, the vehicle speeds corresponding to the simulated and calculated traffic capacities increase as the road adhesion coefficient increases. When the road adhesion coefficient increases from 0.1 to 0.3, under the same length of the upstream transition area, the vehicle speeds corresponding to the simulated and calculated traffic capacities show greater increase than other road adhesion coefficient variation ranges. When the road adhesion coefficient is 0.7 and 0.9, the vehicle speeds corresponding to the simulated and calculated traffic capacities are same for the same length of the upstream transition area.

The calculated traffic capacity *q*_c_ and simulated traffic capacity *q*_s_ and their corresponding vehicle speed limits *v*_qc_ and *v*_qs_ can be expressed with upstream transition area length and road adhesion coefficient by regression method as follows:

$$q_{c}=(176.90ln(\mu )+976.36)(1-exp(-5.53\times 10^{-4}L^{2.89}))(R^{2}=0.9994),$$ (10)

$$q_{s}=(179.65ln(\mu )+819.37)(1-exp(-0.34L^{0.64}))(R^{2}=0.9976),$$ (11)

$$v_{qc}=(14.02ln(\mu )+41.25)(1-exp(-2.03\times 10^{-8}L^{5.88}))(R^{2}=0.9742),$$ (12)

$$v_{qs}=(18.40ln(\mu )+63.55)(1-exp(-0.01L^{1.51}))(R^{2}=0.9903).$$ (13)

**Fig 13:**
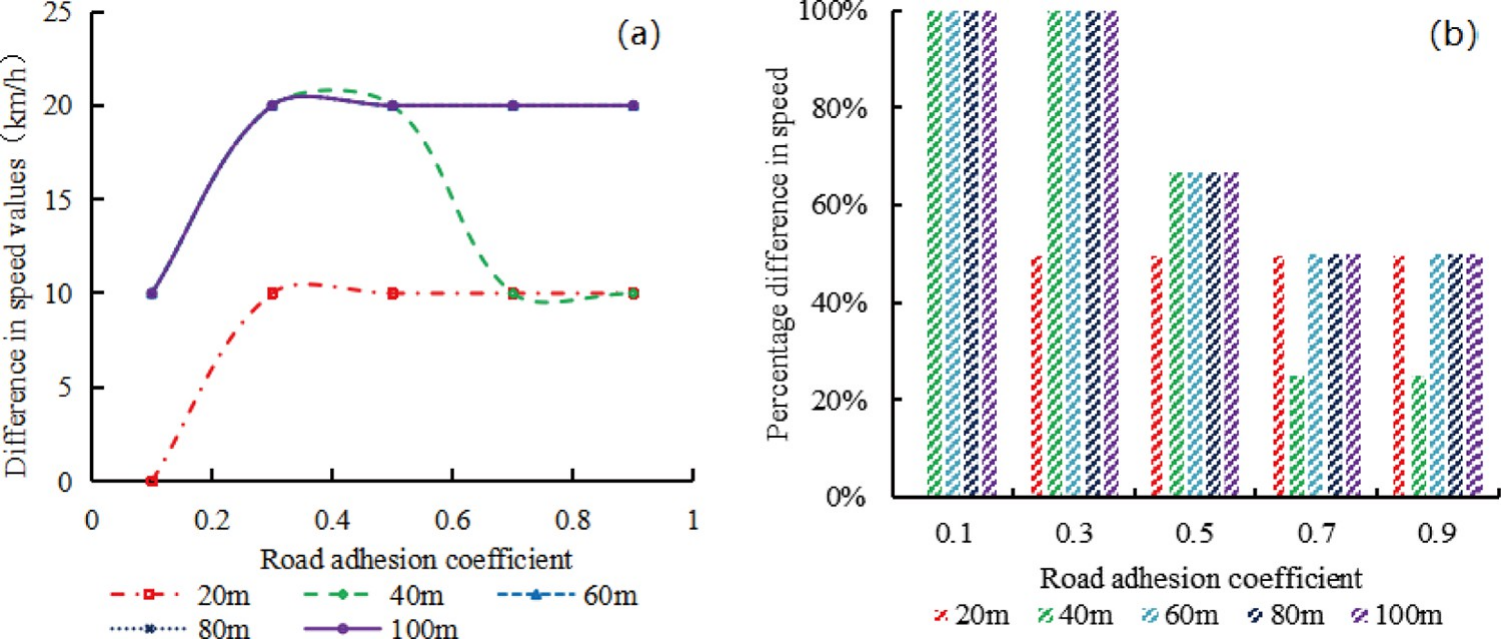
Speed difference between simulated and calculated values in the work zone. (a) Difference in speed limit values; (b) Percentage difference of speed limit values.

The speed limit difference corresponding to the simulated and calculated traffic capacities can be obtained from Fig 11, then the relationship between speed limit differences and the road adhesion coefficient under different length of the upstream transition area can be obtained as shown in Fig 13. Usually, the speed limit corresponding to the simulated traffic capacity exceeds the speed limit corresponding to the calculated value, therefore, the speed limit corresponding to the calculated traffic capacity determined by Formula (8) is not applicable to expressway work zones.

In Fig 13A, when the length of the upstream transition area is 20m, the difference in speed limit is 0 when the road adhesion coefficient is 0.1, and they are 10km/h under other road adhesion conditions. When the length of the upstream transition area is 40m and the road adhesion coefficients are 0.1, 0.3~0.5 and 0.7~0.9, the corresponding speed limit differences are 10km/h, 20km/h and 10km/h, respectively. When the length of the upstream transition area is 60m, 80m, and 100m, the difference in speed limit is 10km/h when the road adhesion coefficient is 0.1, and they are 20km/h for other road adhesion coefficients.

In Fig 13B, when the length of the upstream transition area is 20m, the speed limit corresponds to simulated traffic capacity is 50% higher than the speed limit corresponds to calculated traffic capacity. In other upstream transition area lengths, this percentage decreases on the whole with an increase in road adhesion coefficient, ranging from 100% to 50%.

#### 3.3.3 Traffic conflict rates in a work zone at optimal speed limits

**Fig 14:**
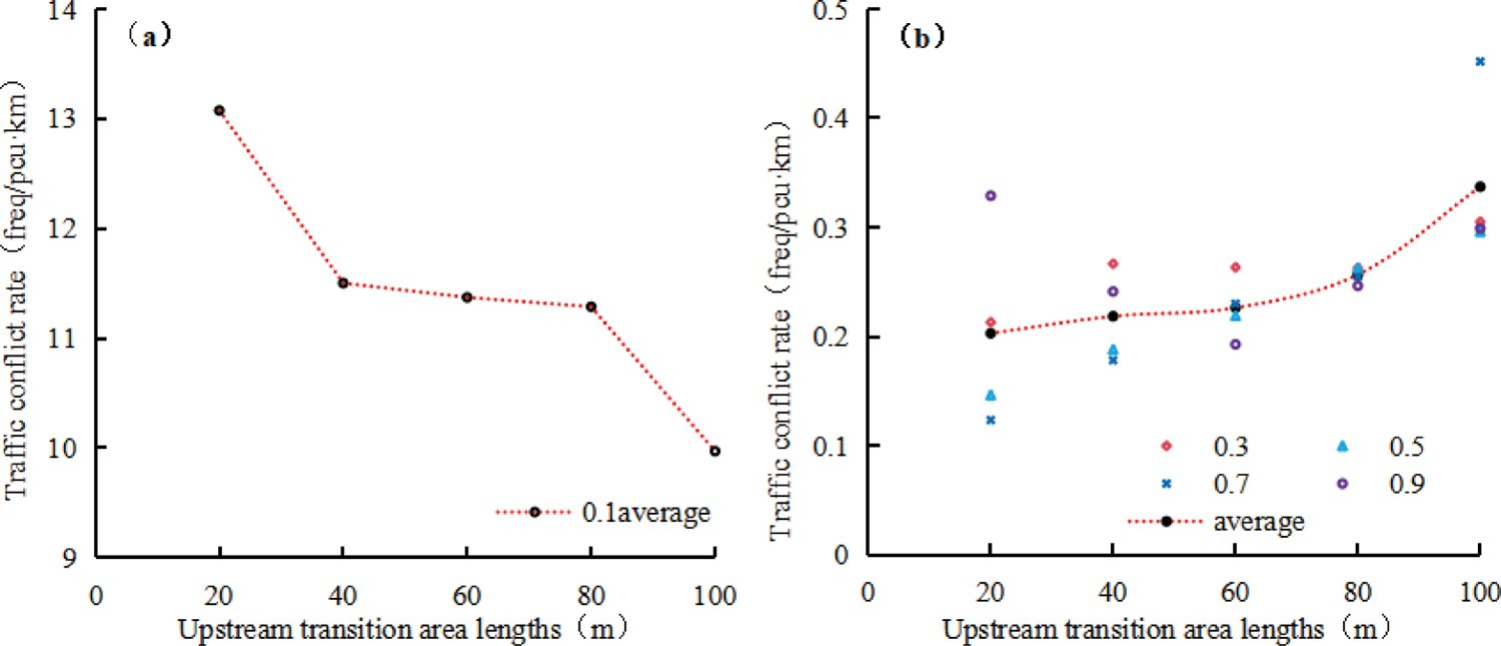
Traffic conflict rate.

The relationships between average traffic conflict rate at optimal speed limits corresponding to the simulated traffic capacity and upstream transition area length under various road adhesion coefficients are depicted in Fig 14. It can be seen from Fig 14 that when the road adhesion coefficient is 0.1, the average traffic conflict rate exceeds 10 freq/(pcu•km). Increasing the length of the upstream transition area can reduce the traffic conflict rate, but the effect is limited, indicating that road closure is a feasible option when the road adhesion coefficient is 0.1. When the road adhesion coefficient is equal to or greater than 0.3, the average conflict rate is lower than 0.5, and with the increase of the length of the upstream transition area, the traffic conflict rate slightly increases.

## 4. Discussion

According to RTSM, the formula for calculating upstream transition area length, when some lanes in the work zone are closed, is as follows:

$$L=\{\begin{array}{cc} v^{2}W/155 & v\leq 60km/h\\ 0.625\times v\cdot W & v\geq 60km/h \end{array},$$ (14)

where *L* is the upstream transition area length, m; *v* is the speed limit of work zone, km/h; *W* is the width of closed lane, m, 3.75 m.

From Formula (14), the speed limit formula expressed with the upstream transition area length can be obtained as follows:

$$v=\{\begin{array}{cc} \sqrt{155L/W} & L\leq 87.10m\\ L/0.625W & L\geq 140.63m \end{array}.$$ (15)

**Fig 15:**
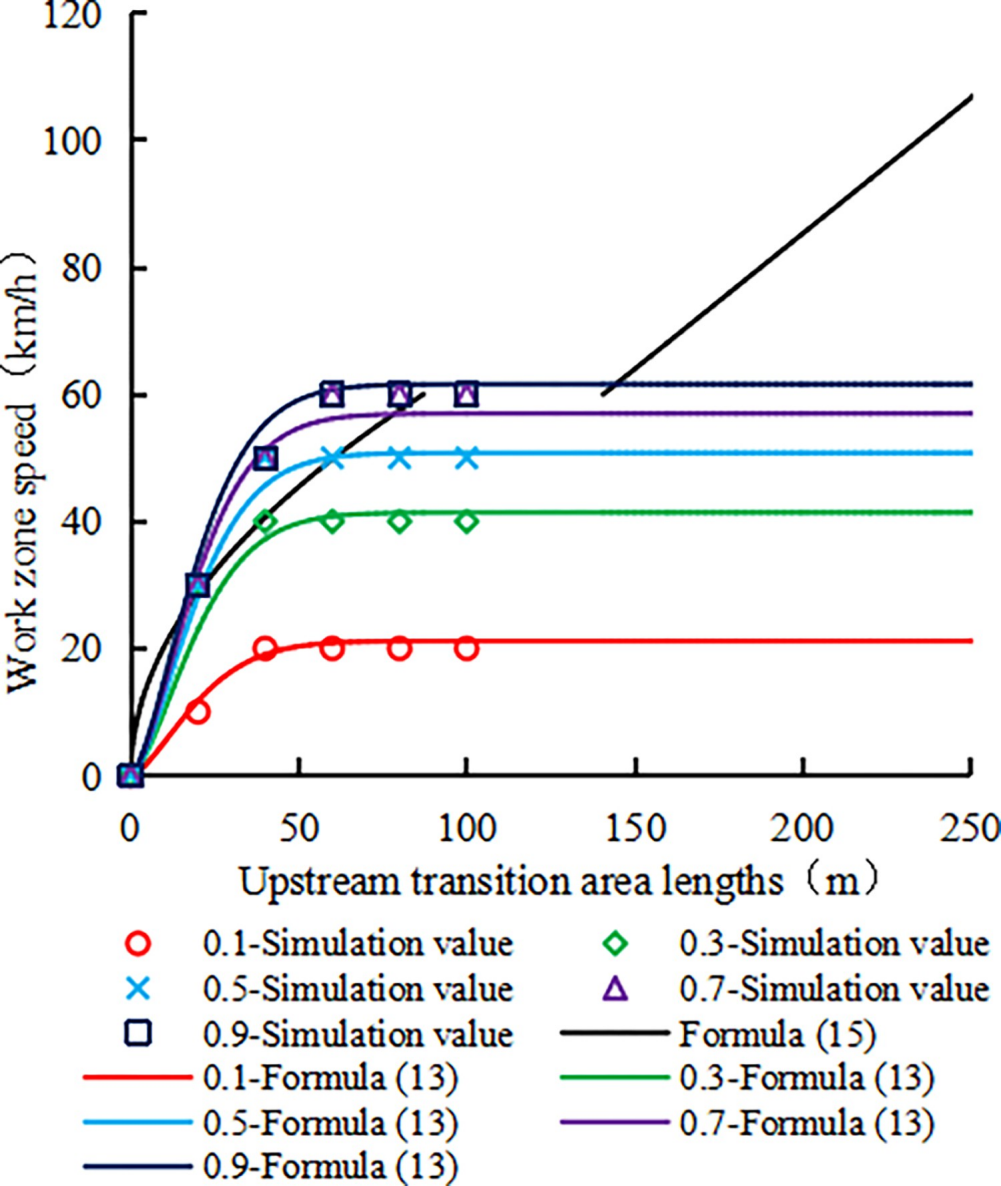
Comparison of Formula (13) with Formula (15).

Formula (15) cannot back-calculate the work zone speed limit when the upstream transition area length is between 87.10 and 140.63 m. Fig 15 shows the work zone speed limits calculated by Formulas (15) and (13) under different upstream transition area lengths and road adhesion coefficients.

In Fig 15, when the road adhesion coefficient is within the range of 0.1 to 0.3, under the same length of the upstream transition area, the speed limit obtained by Formula (15) significantly exceeds that obtained by Formula (13), and the difference between them also increases as the length of the upstream transition area increases. This difference means that the speed limit determined according to Formula (15) is prone to reduce vehicle stability, increase safety risks, and decrease traffic capacity. For road adhesion coefficients of 0.5 ~ 0.9, there are two intersections between the speed limit curves determined by Formulas (15) and (13). Between these two intersections, Formula (15) yields a lower speed limit, improving vehicle driving stability but reducing road traffic capacity. On the contrary, beyond these two intersections, Formula (15) will result in higher speed limits, which is not conducive to improving vehicle driving stability and traffic capacity.

When the length of the upstream transition area exceeds 140.63m, Formula (15) predicts a linear increase in the speed limit value, while Formula (13) remains relatively stable. Increasing the speed limit in Formula (15) will reduce the vehicle driving stability and road traffic capacity, thereby offset the benefits of extending the transition area. In other literature studies, Lei et al. [41] conducted VISSIM simulation analysis based on field observation data and suggested setting the length of the upstream transition area to 90m when the speed limit in the work zone is 60km/h, which can effectively improve road traffic capacity. Meng et al. [42] investigated on-site data from 8 highway work zones and conducted VISSIM simulation analysis, and found that the reasonable length of the upstream transition area should be set between 45-70m. When the speed limit is higher than 50km/h, the number of traffic conflicts will significantly increase with the increase of the speed limit. These studies demonstrate that the proposed speed limit for expressway work zones not exceeding 60km/h is reasonable.

This study emphasizes the quantitative relationship between speed limits in expressway work zones, vehicle driving stability, and road traffic capacity, highlighting the necessity of considering road adhesion coefficient when setting speed limits in expressway work zones. By comparing with RTSM and the research results of other literature, it is found that the speed limit in the expressway work zones determined after considering vehicle driving stability, road traffic capacity, and road adhesion coefficient is safer, more reliable, and more detailed. It is recommended that when setting the speed limit in the expressway work zones, the influence of road surface aging, weather, and construction debris on the road adhesion coefficient must be considered. However, although this paper relatively fully considers the impact of vehicle driving stability and traffic capacity on speed limits when two lanes are changed to one lane in expressway work zones, it does not delve into the situation of different numbers and positions of closed lanes on multi lane roads. In addition, the numerical simulation results are relatively idealized, and the application of research results in engineering practice also requires a lot of practical research. These issues will be the focus of future research to gain a more comprehensive understanding of speed limits in expressway work zones.

The research results can provide scientific basis for the speed limit in the expressway work zones, thus improving traffic safety and efficiency. It can be integrated into a traffic management system, which automatically adjusts the upstream transition area lengths and the speed limits through real-time monitoring of the road adhesion coefficient and the traffic flow, so as to realize the automation and intelligence of the traffic control scheme in the work zone.

## 5. Conclusions

(1) CarSim and TruckSim were utilized to assess the driving stability of cars and trucks across varied upstream transition area lengths, road adhesion coefficients, and speed values within expressway work zones. Critical safe speeds for cars and trucks were determined based on evaluation indices including lateral acceleration, trajectory deviation, and lateral load transfer ratio. The analysis revealed that, with a fixed upstream transition area length, both car and truck critical safe speeds increase exponentially with rising road adhesion coefficients. Conversely, at a constant road adhesion coefficient, critical safe speeds for cars and trucks exhibit a power function relationship with increasing upstream transition area lengths.

(2) VISSIM simulation software was utilized to study the traffic capacity of expressway work zones under varying upstream transition area lengths, road adhesion coefficients, and speed limits. A correlation between traffic capacity, along with its corresponding speed limit and the upstream transition area length, road adhesion coefficient is established. When the upstream transition area length is fixed, both traffic capacity and its corresponding speed limit increase logarithmically with the road adhesion coefficient. Meanwhile, with a constant road adhesion coefficient, traffic capacity and its corresponding speed limit increase exponentially with the upstream transition area length.

(3) When the road adhesion coefficient is 0.1 and 0.3, the speed limit determined by RTMS for an expressway work zone is higher under the same upstream transition area length. When the road adhesion coefficient is greater than or equal to 0.5, there are two intersection points between the speed limit curve determined by RTMS and the simulated speed limit curve in the expressway work zones. Under the upstream transition area length outside these two intersection points, the speed limit determined by RTMS in the expressway work zone is relatively high, and the stability of vehicle driving is poor; Under the upstream transition area length within these two intersections, the speed limit determined by RTMS for the expressway work zone is relatively low. Although lower speed limits can improve vehicle stability, they can also reduce the traffic capacity of expressway work zones.

## Supporting information

S1 Table Relationship between evaluation indexes and influencing factors for car. (a) Lateral acceleration; (b) Trajectory deviation value; (c) Lateral load transfer ratio.
(PDF)

S2 Table The relationship between the critical safe speed and the length of the upstream transition area and the road adhesion coefficient for cars. (PDF)

S3 Table Relationship between evaluation indexes and influencing factors for truck. (a) Lateral acceleration; (b) Trajectory deviation value; (c) Lateral load transfer ratio.
(PDF)

S4 Table The relationship between the critical safe speed and the length of the upstream transition area and the road adhesion coefficient for trucks. (PDF)

S5 Table Maximum design speed. (PDF)

S6 Table Relationship between the simulated and calculated traffic capacity and input traffic volume. (a) L = 20m; (b) L = 40m; (c) L = 60m; (d) L = 80m; (e) L = 100m.
(PDF)

S7 Table Relationship between the calculated and simulated traffic capacities and the speed limit in the work zone. (a) L = 20m; (b) L = 40m; (c) L = 60m; (d) L = 80m; (e) L = 100m.
(PDF)

S8 Table The calculated and simulated traffic capacities and their corresponding vehicle speed limits under different upstream transition area length and road adhesion coefficient. (a) qs; (b) qc; (c) vqs; (d) vqc.
(PDF)

S9 Table Speed difference between simulated and calculated values in the work zone. (a) Difference in speed limit values; (b) Percentage difference of speed limit values.
(PDF)

S10 Table Traffic conflict rate. (PDF)

S11 Table Comparison of Formula (13) with Formula (15). (PDF)
