# Supplementary material for: Examining driving stability and traffic capacity: A simulation study on appropriate speed limits in expressway work zones
Source: PLoS One. 2025 Jan 24;20(1):e0317690. doi: 10.1371/journal.pone.0317690 (PMC11759355; doi:10.1371/journal.pone.0317690)
Supplement: S1 Table — (a) Lateral acceleration; (b) Trajectory deviation value; (c) Lateral load transfer ratio. (PDF) [file pone.0317690.s001.pdf]

S1 Table. Relationship between evaluation indexes and influencing factors for car.

S1 (a) Lateral acceleration

Speed (km/h)	0.1<br>20-0.1	0.1<br>40-0.1	0.1<br>60-0.1	0.1<br>80-0.1	0.1<br>100-0.1	0.3<br>20-0.3	0.3<br>40-0.3	0.3<br>60-0.3	0.3<br>80-0.3	0.3<br>100-0.3
0	0	0	0	0	0	0	0	0	0	0
20	0.111	0.041	0.019	0.011	0.007	0.132	0.041	0.002	0.011	0.007
40	0.107	0.114	0.074	0.044	0.029	0.309	0.152	0.074	0.043	0.028
60	0.114	0.115	0.115	0.115	0.064	0.313	0.302	0.161	0.096	0.063
80	0.113	0.113	0.117	0.113	0.116	0.314	0.319	0.269	0.169	0.112
100	0.112	0.112	0.112	0.112	0.112	0.313	0.316	0.313	0.252	0.180
120	0.114	0.108	0.108	0.109	0.109	0.311	0.312	0.313	0.299	0.247
140	0.098	0.081	0.075	0.071	0.092	0.309	0.309	0.308	0.309	0.288
160	0.060	0.052	0.038	0.026	0.020	0.307	0.307	0.306	0.306	0.302

Speed (km/h)	0.5<br>20-0.5	0.5<br>40-0.5	0.5<br>60-0.5	0.5<br>80-0.5	0.5<br>100-0.5	0.7<br>20-0.7	0.7<br>40-0.7	0.7<br>60-0.7	0.7<br>80-0.7	0.7<br>100-0.7
0	0	0	0	0	0	0	0	0	0	0
20	0.131	0.041	0.020	0.020	0.007	0.131	0.041	0.019	0.011	0.007
40	0.408	0.151	0.074	0.043	0.028	0.424	0.151	0.074	0.043	0.028
60	0.506	0.308	0.161	0.096	0.063	0.621	0.311	0.160	0.096	0.063
80	0.506	0.451	0.273	0.168	0.112	0.646	0.467	0.273	0.168	0.111
100	0.500	0.492	0.373	0.260	0.179	0.649	0.556	0.380	0.260	0.179
120	0.492	0.492	0.435	0.340	0.255	0.649	0.600	0.461	0.346	0.255
140	0.480	0.481	0.455	0.388	0.321	0.646	0.617	0.517	0.404	0.325
160	0.454	0.458	0.444	0.402	0.364	0.640	0.620	0.551	0.448	0.376

Speed (km/h)	0.9<br>20-0.9	0.9<br>40-0.9	0.9<br>60-0.9	0.9<br>80-0.9	0.9<br>100-0.9
0	0	0	0	0	0

20	0.131	0.041	0.019	0.011	0.007
40	0.427	0.155	0.074	0.043	0.028
60	0.697	0.311	0.160	0.096	0.063
80	0.793	0.478	0.273	0.168	0.112
100	0.812	0.597	0.383	0.261	0.179
120	0.813	0.671	0.474	0.347	0.255
140	0.805	0.710	0.546	0.410	0.326
160	0.790	0.726	0.624	0.462	0.379

S1 (b)Trajectory deviation

Speed（km/h）	0.1 20-0.1	0.1 40-0.1	0.1 60-0.1	0.1 80-0.1	0.1 100-0.1	0.3 20-0.3	0.3 40-0.3	0.3 60-0.3	0.3 80-0.3	0.3 100-0.3
0	0	0	0	0	0	0	0	0	0	0
20	0.770	0.046	0.023	0.014	0.010	0.146	0.046	0.023	0.014	0.010
40	2.506	0.899	0.062	0.039	0.028	0.563	0.121	0.063	0.039	0.028
60	3.028	2.042	1.002	0.190	0.042	1.657	0.183	0.096	0.062	0.044
80	3.270	2.644	1.887	1.112	0.462	2.224	0.784	0.133	0.074	0.051
100	3.395	2.958	2.421	1.836	1.256	2.534	1.389	0.490	0.165	0.083
120	3.504	3.222	2.870	2.463	2.021	2.760	1.854	0.976	0.416	0.193
140	3.594	3.445	3.279	3.098	2.902	2.919	2.199	1.439	0.804	0.421
160	3.647	3.599	3.591	3.630	3.705	3.040	2.475	1.851	1.245	0.760

Speed（km/h）	0.5 20-0.5	0.5 40-0.5	0.5 60-0.5	0.5 80-0.5	0.5 100-0.5	0.7 20-0.7	0.7 40-0.7	0.7 60-0.7	0.7 80-0.7	0.7 100-0.7
0	0	0	0	0	0	0	0	0	0	0
20	0.146	0.046	0.023	0.014	0.010	0.146	0.046	0.023	0.014	0.010
40	0.316	0.123	0.064	0.039	0.028	0.342	0.123	0.064	0.039	0.028
60	0.770	0.182	0.098	0.062	0.044	0.531	0.185	0.099	0.062	0.044
80	1.397	0.303	0.127	0.075	0.052	0.964	0.271	0.128	0.076	0.052
100	1.813	0.658	0.241	0.122	0.075	1.352	0.500	0.218	0.118	0.073

120	2.120	1.056	0.461	0.233	0.142	1.672	0.788	0.380	0.213	0.135
140	2.341	1.419	0.759	0.417	0.261	1.922	1.087	0.603	0.363	0.241
160	2.514	1.736	1.087	0.658	0.424	2.122	1.377	0.858	0.551	0.381

	0.9	0.9	0.9	0.9	0.9	0.9
Speed（km/h）	20-0.9	40-0.9	60-0.9	80-0.9	100-0.9	20-0.9
0	0	0	0	0	0	0
20	0.147	0.046	0.023	0.014	0.010	0.147
40	0.348	0.123	0.064	0.039	0.028	0.348
60	0.485	0.186	0.099	0.062	0.044	0.485
80	0.800	0.268	0.128	0.076	0.052	0.800
100	1.138	0.453	0.213	0.116	0.073	1.138
120	1.438	0.696	0.360	0.207	0.132	1.438
140	1.691	0.964	0.559	0.348	0.234	1.691
160	1.902	1.229	1.023	0.522	0.368	1.902

S1 (c)Lateral load transfer ratio

	0.1	0.1	0.1	0.1	0.1	0.3	0.3	0.3	0.3	0.3
Speed（km/h）	20-0.1	40-0.1	60-0.1	80-0.1	100-0.1	20-0.3	40-0.3	60-0.3	80-0.3	100-0.3
0	0	0	0	0	0	0	0	0	0	0
20	0.065	0.044	0.028	0.022	0.022	0.109	0.044	0.028	0.022	0.019
40	0.070	0.069	0.068	0.046	0.035	0.211	0.125	0.068	0.046	0.035
60	0.077	0.076	0.076	0.070	0.061	0.225	0.210	0.132	0.084	0.060
80	0.083	0.075	0.087	0.077	0.082	0.226	0.243	0.196	0.137	0.096
100	0.086	0.076	0.075	0.077	0.076	0.226	0.235	0.226	0.182	0.139
120	0.088	0.074	0.074	0.075	0.075	0.222	0.223	0.231	0.210	0.175
140	0.089	0.072	0.067	0.062	0.072	0.220	0.217	0.219	0.217	0.199
160	0.095	0.070	0.055	0.044	0.038	0.222	0.216	0.214	0.212	0.210

	0.5	0.5	0.5	0.5	0.5	0.7	0.7	0.7	0.7	0.7
Speed	20-	40-	60-	80-	100-	20-	40-	60-	80-	100-

（km/h）	0.5	0.5	0.5	0.5	0.5	0.7	0.7	0.7	0.7	0.7
0	0	0	0	0	0	0	0	0	0	0
20	0.109	0.044	0.028	0.022	0.019	0.109	0.044	0.028	0.022	0.019
40	0.314	0.124	0.068	0.046	0.035	0.328	0.124	0.068	0.046	0.035
60	0.381	0.241	0.132	0.084	0.060	0.472	0.243	0.131	0.084	0.060
80	0.384	0.318	0.187	0.137	0.096	0.496	0.361	0.212	0.136	0.096
100	0.380	0.370	0.279	0.196	0.140	0.502	0.428	0.292	0.197	0.140
120	0.373	0.372	0.320	0.250	0.187	0.502	0.462	0.357	0.256	0.187
140	0.359	0.365	0.339	0.289	0.230	0.498	0.477	0.401	0.309	0.234
160	0.348	0.360	0.346	0.314	0.265	0.493	0.483	0.428	0.351	0.277

	0.9	0.9	0.9	0.9	0.9
Speed（km/h）	20-0.9	40-0.9	60-0.9	80-0.9	100-0.9
0	0	0	0	0	0
20	0.109	0.044	0.028	0.022	0.019
40	0.330	0.124	0.068	0.046	0.035
60	0.533	0.243	0.131	0.084	0.060
80	0.606	0.369	0.213	0.136	0.096
100	0.622	0.460	0.295	0.197	0.149
120	0.624	0.500	0.368	0.258	0.188
140	0.619	0.531	0.424	0.314	0.236
160	0.610	0.565	0.488	0.362	0.280
