# Supplementary material for: Examining driving stability and traffic capacity: A simulation study on appropriate speed limits in expressway work zones
Source: PLoS One. 2025 Jan 24;20(1):e0317690. doi: 10.1371/journal.pone.0317690 (PMC11759355; doi:10.1371/journal.pone.0317690)
Supplement: S2 Table — (PDF) [file pone.0317690.s002.pdf]

S2 Table. The relationship between the critical safe speed and the length of the upstream transition area and the road adhesion coefficient for cars.

	0	0.1	0.3	0.5	0.7	0.9
simulation value-20m	0	13.5	31.9	37.7	35.5	35
simulation value-40m	0	32.6	65	72.8	70.8	70.3
simulation value-60m	0	46.7	91.9	106.3	104.2	103.4
simulation value-80m	0	62.3	113.3	129	132.8	134.1
simulation value-100m	0	75.5	131.7	145.5	149.3	150.7
fitting value-20m	0	19.09	35.31	39.26	40.14	40.33
fitting value-40m	0	33.94	62.76	69.79	71.36	71.70
fitting value-60m	0	47.51	87.87	97.71	99.92	100.39
fitting value-80m	0	60.33	111.57	124.07	126.86	127.46
fitting value-100m	0	72.60	134.27	149.31	152.68	153.40
