# Supplementary material for: Examining driving stability and traffic capacity: A simulation study on appropriate speed limits in expressway work zones
Source: PLoS One. 2025 Jan 24;20(1):e0317690. doi: 10.1371/journal.pone.0317690 (PMC11759355; doi:10.1371/journal.pone.0317690)
Supplement: S3 Table — (a) Lateral acceleration; (b) Trajectory deviation value; (c) Lateral load transfer ratio. (PDF) [file pone.0317690.s003.pdf]

S3 Table. Relationship between evaluation indexes and influencing factors for truck.

S3 (a) Lateral acceleration

	0.1	0.1	0.1	0.1	0.1	0.3	0.3	0.3	0.3	0.3
Speed （km/h）	20-0.1	40-0.1	60-0.1	80-0.1	100-0.1	20-0.3	40-0.3	60-0.3	80-0.3	100-0.3
0	0	0	0	0	0	0	0	0	0	0
20	0.086	0.037	0.020	0.011	0.007	0.115	0.037	0.020	0.011	0.007
40	0.087	0.110	0.069	0.041	0.028	0.261	0.127	0.067	0.041	0.027
60	0.086	0.097	0.114	0.104	0.060	0.283	0.220	0.137	0.088	0.060
80	0.127	0.129	0.132	0.132	0.074	0.282	0.269	0.214	0.151	0.106
100	0.158	0.157			0.156	0.287	0.279	0.271	0.220	0.163
120						0.296	0.293	0.291	0.269	0.223
140						0.322	0.319	0.315	0.295	0.257
160						0.348	0.345	0.338	0.321	0.291

	0.5	0.5	0.5	0.5	0.5	0.7	0.7	0.7	0.7	0.7
Speed （km/h）	20-0.5	40-0.5	60-0.5	80-0.5	100-0.5	20-0.7	40-0.7	60-0.7	80-0.7	100-0.7
0	0	0	0	0	0	0	0	0	0	0
20	0.115	0.037	0.020	0.020	0.007	0.115	0.037	0.018	0.011	0.007
40	0.288	0.127	0.067	0.041	0.027	0.288	0.127	0.067	0.041	0.027
60	0.363	0.223	0.136	0.088	0.060	0.372	0.223	0.135	0.088	0.060
80	0.382	0.290	0.210	0.149	0.105	0.396	0.293	0.208	0.149	0.105
100	0.389	0.329	0.273	0.214	0.160	0.403	0.336	0.272	0.212	0.160
120	0.394	0.360	0.322	0.269	0.216	0.409	0.366	0.321	0.268	0.214
140	0.412	0.387	0.352	0.307	0.256	0.428	0.396	0.356	0.306	0.255
160	0.431	0.413	0.383	0.345	0.297	0.448	0.426	0.390	0.345	0.296

	0.9	0.9	0.9	0.9	0.9
Speed （km/h）	20-0.9	40-0.9	60-0.9	80-0.9	100-0.9
0	0	0	0	0	0

20	0.115	0.037	0.020	0.020	0.007
40	0.288	0.127	0.067	0.041	0.027
60	0.373	0.223	0.135	0.088	0.060
80	0.399	0.295	0.208	0.149	0.105
100	0.408	0.339	0.257	0.212	0.160
120	0.414	0.366	0.321	0.267	0.214
140	0.432	0.396	0.356	0.307	0.255
160	0.451	0.427	0.390	0.346	0.296

S3 (b) Trajectory deviation value

	0.1	0.1	0.1	0.1	0.1	0.3	0.3	0.3	0.3	0.3
Speed （km/h）	20-0.1	40-0.1	60-0.1	80-0.1	100-0.1	20-0.3	40-0.3	60-0.3	80-0.3	100-0.3
0	0	0	0	0	0	0	0	0	0	0
20	3.466	0.093	0.050	0.032	0.023	0.276	0.093	0.050	0.032	0.023
40	2.525	0.989	0.098	0.063	0.044	0.442	0.187	0.100	0.063	0.044
60	2.913	2.006	0.944	0.106	0.063	1.055	0.320	0.156	0.093	0.063
80					0.535	1.477	0.585	0.265	0.141	0.087
100						1.727	0.903	0.456	0.240	0.140
120						1.891	1.157	0.664	0.382	0.226
140						1.965	1.313	0.825	0.511	0.321
160						2.038	1.469	0.985	0.640	0.415

	0.5	0.5	0.5	0.5	0.5	0.7	0.7	0.7	0.7	0.7
Speed （km/h）	20-0.5	40-0.5	60-0.5	80-0.5	100-0.5	20-0.7	40-0.7	60-0.7	80-0.7	100-0.7
0	0	0	0	0	0	0	0	0	0	0
20	0.277	0.093	0.050	0.032	0.023	0.277	0.093	0.050	0.032	0.023
40	0.487	0.187	0.100	0.063	0.044	0.496	0.188	0.100	0.063	0.044
60	0.741	0.315	0.157	0.093	0.063	0.724	0.314	0.157	0.093	0.063
80	1.043	0.509	0.257	0.141	0.088	1.002	0.500	0.255	0.141	0.088
100	1.280	0.725	0.403	0.229	0.138	1.225	0.703	0.395	0.227	0.138

120	1.460	0.918	0.559	0.342	0.212	1.400	0.884	0.544	0.334	0.211
140	1.565	1.055	0.689	0.447	0.293	1.506	1.016	0.668	0.435	0.286
160	1.669	1.191	0.818	0.552	0.373	1.612	1.147	0.792	0.536	0.361

	0.9	0.9	0.9	0.9	0.9
Speed（km/h）	20-0.9	40-0.9	60-0.9	80-0.9	100-0.9
0	0	0	0	0	0
20	0.277	0.093	0.050	0.032	0.023
40	0.500	0.188	0.100	0.063	0.044
60	0.719	0.314	0.157	0.093	0.063
80	0.990	0.496	0.255	0.141	0.088
100	1.209	0.694	0.392	0.226	0.138
120	1.382	0.872	0.537	0.332	0.210
140	1.488	1.002	0.658	0.430	0.284
160	1.594	1.132	0.779	0.528	0.359

S3 (c) Lateral load transfer ratio

	0.1	0.1	0.1	0.1	0.1	0.3	0.3	0.3	0.3	0.3
Speed（km/h）	20-0.1	40-0.1	60-0.1	80-0.1	100-0.1	20-0.3	40-0.3	60-0.3	80-0.3	100-0.3
0	0	0	0	0	0	0	0	0	0	0
20	0.139	0.063	0.042	0.033	0.031	0.147	0.063	0.042	0.033	0.031
40	0.134	0.110	0.098	0.068	0.052	0.311	0.165	0.097	0.068	0.052
60	0.130	0.126	0.117	0.110	0.088	0.317	0.269	0.172	0.119	0.088
80	0.271	0.272	0.277	0.277	0.188	0.310	0.313	0.240	0.175	0.132
100		0.407		0.460	0.460	0.301	0.312	0.278	0.222	0.174
120						0.308	0.305	0.294	0.283	0.211
140						0.311	0.308	0.297	0.282	0.232
160						0.314	0.311	0.300	0.280	0.252
	0.5	0.5	0.5	0.5	0.5	0.7	0.7	0.7	0.7	0.7
Speed	20-0.5	40-0.5	60-0.5	80-0.5	100-	20-0.7	40-0.7	60-0.7	80-0.7	100-

（km/h）					0.5					0.7
0	0	0	0	0	0	0	0	0	0	0
20	0.147	0.063	0.042	0.033	0.031	0.147	0.063	0.042	0.033	0.031
40	0.345	0.164	0.097	0.068	0.052	0.346	0.164	0.097	0.068	0.052
60	0.431	0.272	0.173	0.119	0.088	0.442	0.273	0.173	0.119	0.088
80	0.447	0.346	0.244	0.176	0.132	0.467	0.349	0.245	0.176	0.132
100	0.446	0.380	0.296	0.227	0.176	0.470	0.391	0.300	0.228	0.176
120	0.440	0.397	0.329	0.265	0.213	0.465	0.412	0.337	0.269	0.214
140	0.430	0.396	0.371	0.286	0.239	0.454	0.413	0.353	0.292	0.241
160	0.421	0.396	0.413	0.308	0.265	0.443	0.415	0.369	0.315	0.268

	0.9	0.9	0.9	0.9	0.9
Speed（km/h）	20-0.9	40-0.9	60-0.9	80-0.9	100-0.9
0	0	0	0	0	0
20	0.147	0.063	0.042	0.031	0.031
40	0.346	0.164	0.097	0.068	0.052
60	0.445	0.273	0.173	0.119	0.088
80	0.472	0.351	0.246	0.177	0.132
100	0.475	0.394	0.302	0.229	0.177
120	0.471	0.415	0.339	0.270	0.215
140	0.460	0.417	0.356	0.293	0.242
160	0.450	0.419	0.372	0.317	0.268
