# Supplementary material for: Examining driving stability and traffic capacity: A simulation study on appropriate speed limits in expressway work zones
Source: PLoS One. 2025 Jan 24;20(1):e0317690. doi: 10.1371/journal.pone.0317690 (PMC11759355; doi:10.1371/journal.pone.0317690)
Supplement: S4 Table — (PDF) [file pone.0317690.s004.pdf]

S4 Table. The relationship between the critical safe speed and the length of the upstream transition area and the road adhesion coefficient for trucks.

	0	0.1	0.3	0.5	0.7	0.9
simulation value-20m	0	10.20	23.00	22.20	22.10	22.10
simulation value-40m	0	24.60	57.00	57.70	57.80	57.80
simulation value-60m	0	44.80	83.70	85.90	86.40	86.50
simulation value-80m	0	65.30	108.40	112.60	113.70	113.90
simulation value-100m	0	69.50	135.60	141.80	143.80	144.20
fitting value-20m	0	13.75	25.83	27.00	27.05	27.06
fitting value-40m	0	28.26	53.11	55.51	55.63	55.63
fitting value-60m	0	43.09	80.96	84.63	84.81	84.81
fitting value-80m	0	58.12	109.20	114.14	114.38	114.39
fitting value-100m	0	73.30	137.72	143.96	144.26	144.27
