# Supplementary material for: Examining driving stability and traffic capacity: A simulation study on appropriate speed limits in expressway work zones
Source: PLoS One. 2025 Jan 24;20(1):e0317690. doi: 10.1371/journal.pone.0317690 (PMC11759355; doi:10.1371/journal.pone.0317690)
Supplement: S5 Table — (PDF) [file pone.0317690.s005.pdf]

S5 Table. Maximum design speed

	0.1	0.3	0.5	0.7	0.9
L=20m	10	20	20	20	20
L=40m	20	50	50	50	50
L=60m	40	80	80	80	80
L=80m	60	100	110	110	110
L=100m	60	120	120	120	120
