# Supplementary material for: Examining driving stability and traffic capacity: A simulation study on appropriate speed limits in expressway work zones
Source: PLoS One. 2025 Jan 24;20(1):e0317690. doi: 10.1371/journal.pone.0317690 (PMC11759355; doi:10.1371/journal.pone.0317690)
Supplement: S6 Table — (a) L = 20m; (b) L = 40m; (c) L = 60m; (d) L = 80m; (e) L = 100m. (PDF) [file pone.0317690.s006.pdf]

S6 Table. Relationship between the simulated and calculated traffic capacity and input traffic volume.

S6 (a) L=20m

	0.1-10km/h		0.3-20km/h		0.5-20km/h	
	calculated value	simulation value	calculated value	simulation value	calculated value	simulation value
200	559	141	765	155	832	154
400	559	287	765	340	832	339
600	559	303	765	509	832	510
800	559	304	765	537	832	565
1000	559	301	765	534	832	569
1200	559	300	765	539	832	569
1400	559	300	765	539	832	569

	0.7-20km/h		0.9-20km/h	
	calculated value	simulation value	calculated value	simulation value
200	864	155	883	155
400	864	339	883	339
600	864	512	883	517
800	864	583	883	656
1000	864	588	883	671
1200	864	588	883	675
1400	864	588	883	675

S6 (b) L=40m

	0.1-20km/h		0.3-40km/h		0.5-50km/h	
	calculated value	simulation value	calculated value	simulation value	calculated value	simulation value
200	546	161	717	166	814	168
400	546	345	717	356	814	359

600	546	413	717	544	814	554
800	546	412	717	598	814	682
1000	546	413	717	601	814	694
1200	546	414	717	601	814	695
1400	546	414	717	602	814	695

	0.7-50km/h		0.9-50km/h	
	calculated value	simulation value	calculated value	simulation value
200	894	167	950	168
400	894	359	950	359
600	894	588	950	561
800	894	727	950	746
1000	894	723	950	754
1200	894	723	950	758
1400	894	722	950	758

S6 (c) L=60m

	0.1-20km/h		0.3-40km/h		0.5-50km/h	
	calculated value	simulation value	calculated value	simulation value	calculated value	simulation value
200	546	160	717	166	814	169
400	546	343	717	357	814	361
600	546	413	717	546	814	557
800	546	413	717	600	814	663
1000	546	413	717	602	814	696
1200	546	413	717	602	814	685
1400	546	414	717	602	814	695

	0.7-60km/h		0.9-60km/h	
	calculated value	simulation value	calculated value	simulation value
200	873	171	931	171

400	873	365	931	365
600	873	566	931	569
800	873	732	931	753
1000	873	754	931	802
1200	873	756	931	806
1400	873	756	931	806

S6 (d) L=80m

	0.1-20km/h		0.3-40km/h		0.5-50km/h	
	calculated value	simulation value	calculated value	simulation value	calculated value	simulation value
200	546	161	717	166	814	170
400	546	343	717	357	814	361
600	546	414	717	547	814	558
800	546	414	717	602	814	683
1000	546	414	717	603	814	696
1200	546	415	717	604	814	696
1400	546	415	717	604	814	696

	0.7-60km/h		0.9-60km/h	
	calculated value	simulation value	calculated value	simulation value
200	873	171	931	171
400	873	365	931	365
600	873	566	931	569
800	873	732	931	752
1000	873	754	931	802
1200	873	756	931	806
1400	873	756	931	806

S6 (e) L=100m

	0.1-20km/h		0.3-40km/h		0.5-50km/h	

	calculated value	simulation value	calculated value	simulation value	calculated value	simulation value
200	546	159	717	165	814	167
400	546	342	717	356	814	359
600	546	412	717	534	814	554
800	546	411	717	597	814	681
1000	546	411	717	600	814	692
1200	546	411	717	600	814	693
1400	546	412	717	600	814	693

	0.7-60km/h		0.9-60km/h	
	calculated value	simulation value	calculated value	simulation value
200	873	170	931	170
400	873	364	931	364
600	873	564	931	567
800	873	730	931	751
1000	873	752	931	801
1200	873	754	931	805
1400	873	754	931	804
