# Supplementary material for: Examining driving stability and traffic capacity: A simulation study on appropriate speed limits in expressway work zones
Source: PLoS One. 2025 Jan 24;20(1):e0317690. doi: 10.1371/journal.pone.0317690 (PMC11759355; doi:10.1371/journal.pone.0317690)
Supplement: S7 Table — (a) L = 20m; (b) L = 40m; (c) L = 60m; (d) L = 80m; (e) L = 100m. (PDF) [file pone.0317690.s007.pdf]

S7 Table. Relationship between the calculated and simulated traffic capacities and the speed limit in the work zone.

S7 (a) L=20m

Speed(km/h)	u=0.1 simulation	u=0.3simulation	u=0.5simulation	u=0.7simulation	u=0.9simulation
10	301	345	359	356	424
20		534	569	588	671
**Speed(km/h)**	**u=0.1calculate**	**u=0.3calculate**	**u=0.5calculate**	**u=0.7calculate**	**u=0.9calculate**
10	559	655	679	689	695
20		765	832	864	883

S7 (b) L=40m

Speed(km/h)	u=0.1 simulation	u=0.3simulation	u=0.5simulation	u=0.7simulation	u=0.9simulation
10	390	326	331	329	338
20	413	534	570	585	602
30		593	657	689	714
40		601	692	707	729
50		583	694	723	754
**Speed**	**u=0.1calculate**	**u=0.3calculate**	**u=0.5calculate**	**u=0.7calculate**	**u=0.9calculate**
10	559	655	679	689	695
20	546	765	832	864	883
30		757	859	912	944
40		717	844	914	957
50		671	814	873	950

S7 (c) L=60m

Speed(km/h)	u=0.1 simulation	u=0.3simulation	u=0.5simulation	u=0.7simulation	u=0.9simulation
10	301	326	336	342	347
20	413	536	575	592	723
30	402	595	662	692	762
40	368	602	694	739	780
50		584	696	753	794

60		560	681	754	802
70		533	669	744	801
80		513	646	731	793

Speed(km/h)	u=0.1calculate	u=0.3calculate	u=0.5calculate	u=0.7calculate	u=0.9calculate
10	559	655	679	689	695
20	546	765	832	864	883
30	474	757	859	912	944
40	409	717	844	914	957
50		671	814	894	950
60		626	779	873	931
70		584	743	842	909
80		546	708	812	883

S7 (d) L=80m

Speed(km/h)	u=0.1 simulation	u=0.3simulation	u=0.5simulation	u=0.7simulation	u=0.9simulation
10	301	326	327	340	345
20	414	538	573	591	605
30	401	597	660	691	714
40	367	603	695	738	770
50		583	696	750	794
60		560	681	753	802
70		533	663	745	800
80		514	645	731	788
100			609	699	765

Speed(km/h)	u=0.1calculate	u=0.3calculate	u=0.5calculate	u=0.7calculate	u=0.9calculate
10	559	655	679	689	695
20	546	765	832	864	883
30	474	757	859	912	944
40	409	717	844	914	957
50		671	814	894	950
60		626	779	873	931

70		584	743	842	909
80		546	708	812	883
100			644	754	832

S7 (e) L=100m

Speed(km/h)	u=0.1 simulation	u=0.3simulation	u=0.5simulation	u=0.7simulation	u=0.9simulation
10	299	320	320	330	336
20	411	532	567	524	600
40	367	600	690	736	768
50		579	692	745	792
60		558	680	752	801
70		533	653	744	798
80		514	645	729	792
100		460	609	697	765
120		315	573	665	756

Speed(km/h)	u=0.1calculate	u=0.3calculate	u=0.5calculate	u=0.7calculate	u=0.9calculate
10	559	655	679	689	695
20	546	765	832	864	883
40	409	717	844	914	957
50		671	814	894	950
60		626	779	873	931
70		584	743	842	909
80		546	708	812	883
100		482	644	754	832
120		430	589	700	783
