# Supplementary material for: Examining driving stability and traffic capacity: A simulation study on appropriate speed limits in expressway work zones
Source: PLoS One. 2025 Jan 24;20(1):e0317690. doi: 10.1371/journal.pone.0317690 (PMC11759355; doi:10.1371/journal.pone.0317690)
Supplement: S8 Table — (a) qs; (b) qc; (c) vqs; (d) vqc. (PDF) [file pone.0317690.s008.pdf]

S8 Table. The calculated and simulated traffic capacities and their corresponding vehicle speed limits under different upstream transition area length and road adhesion coefficient.

S8 (a) q_s

	0.1	0.3	0.5	0.7	0.9
0m	0	0	0	0	0
20m	300	590	650	670	720
40m	410	600	690	720	760
60m	410	600	690	750	800
80m	410	600	690	750	800
100m	410	600	690	750	800

S8 (b) q_c

	0.1	0.3	0.5	0.7	0.9
0m	0	0	0	0	0
20m	559	765	832	864	883
40m	559	765	859	914	957
60m	559	765	859	914	957
80m	559	765	859	914	957
100m	559	765	859	914	957

S8 (c) v_{qs}

	0.1	0.3	0.5	0.7	0.9
0m	0	0	0	0	0
20m	10	30	30	30	30
40m	20	40	50	50	50
60m	20	40	50	60	60
80m	20	40	50	60	60
100m	20	40	50	60	60

S8 (d) v_{qc}

	0.1	0.3	0.5	0.7	0.9

0m	0	0	0	0	0
20m	10	20	20	20	20
40m	10	20	30	40	40
60m	10	20	30	40	40
80m	10	20	30	40	40
100m	10	20	30	40	40
