# Supplementary material for: Examining driving stability and traffic capacity: A simulation study on appropriate speed limits in expressway work zones
Source: PLoS One. 2025 Jan 24;20(1):e0317690. doi: 10.1371/journal.pone.0317690 (PMC11759355; doi:10.1371/journal.pone.0317690)
Supplement: S9 Table — (a) Difference in speed limit values; (b) Percentage difference of speed limit values. (PDF) [file pone.0317690.s009.pdf]

S9 Table. Speed difference between simulated and calculated values in the work zone.

S9 (a) Difference in speed limit values

	0.1	0.3	0.5	0.7	0.9
20m	0	10	10	10	10
40m	10	20	20	10	10
60m	10	20	20	20	20
80m	10	20	20	20	20
100m	10	20	20	20	20

S9 (b) Percentage difference of speed limit values

	0.1	0.3	0.5	0.7	0.9
20m	0%	50%	50%	50%	50%
40m	100%	100%	67%	25%	25%
60m	100%	100%	67%	50%	50%
80m	100%	100%	67%	50%	50%
100m	100%	100%	67%	50%	50%
