# Supplementary material for: Examining driving stability and traffic capacity: A simulation study on appropriate speed limits in expressway work zones
Source: PLoS One. 2025 Jan 24;20(1):e0317690. doi: 10.1371/journal.pone.0317690 (PMC11759355; doi:10.1371/journal.pone.0317690)
Supplement: S10 Table — (PDF) [file pone.0317690.s010.pdf]

S10 Table. Traffic conflict rate.

	0.1average	0.3	0.5	0.7	0.9	average
20m	13.075	0.213	0.146	0.123	0.329	0.203
40m	11.502	0.267	0.188	0.178	0.241	0.218
60m	11.372	0.263	0.219	0.230	0.193	0.226
80m	11.286	0.263	0.263	0.255	0.246	0.257
100m	9.969	0.305	0.296	0.452	0.299	0.337
