# Supplementary material for: Examining driving stability and traffic capacity: A simulation study on appropriate speed limits in expressway work zones
Source: PLoS One. 2025 Jan 24;20(1):e0317690. doi: 10.1371/journal.pone.0317690 (PMC11759355; doi:10.1371/journal.pone.0317690)
Supplement: S11 Table — (PDF) [file pone.0317690.s011.pdf]

S11 Table. Comparison of formula (13) with formula (15).

	0.1	0.3	0.5	0.7	0.9	formula (15)
0.024	0.001	0.001	0.002	0.002	0.002	1
0.097	0.006	0.011	0.013	0.015	0.016	2
0.218	0.019	0.037	0.045	0.050	0.054	3
0.387	0.044	0.087	0.107	0.120	0.129	4
0.605	0.087	0.170	0.209	0.234	0.253	5
0.871	0.151	0.295	0.362	0.406	0.439	6
1.185	0.240	0.468	0.574	0.644	0.697	7
1.548	0.357	0.698	0.857	0.961	1.039	8
1.960	0.508	0.993	1.218	1.367	1.477	9
2.419	0.695	1.358	1.666	1.869	2.021	10
2.927	0.921	1.800	2.209	2.478	2.679	11
3.484	1.189	2.325	2.853	3.201	3.460	12
4.089	1.503	2.937	3.604	4.043	4.371	13
4.742	1.862	3.639	4.466	5.010	5.417	14
5.444	2.269	4.435	5.442	6.105	6.600	15
6.194	2.724	5.324	6.533	7.329	7.924	16
6.992	3.226	6.306	7.739	8.682	9.386	17
7.839	3.776	7.381	9.057	10.161	10.986	18
8.734	4.371	8.544	10.484	11.762	12.716	19
9.677	5.009	9.790	12.013	13.478	14.572	20
10.669	5.686	11.114	13.638	15.300	16.542	21
11.710	6.399	12.507	15.347	17.218	18.615	22
12.798	7.142	13.960	17.130	19.219	20.778	23
13.935	7.911	15.463	18.975	21.288	23.015	24
15.121	8.700	17.005	20.866	23.410	25.310	25
16.355	9.502	18.572	22.790	25.568	27.643	26
17.637	10.311	20.153	24.730	27.744	29.996	27
18.968	11.120	21.735	26.670	29.921	32.349	28

20.000	11.724	22.915	28.119	31.547	34.107	28.754
20.347	11.922	23.303	28.595	32.080	34.684	29
21.774	12.711	24.845	30.487	34.204	36.980	30
23.250	13.481	26.350	32.334	36.275	39.219	31
24.774	14.225	27.805	34.119	38.278	41.385	32
26.347	14.939	29.200	35.831	40.199	43.461	33
27.968	15.618	30.527	37.459	42.025	45.436	34
29.637	16.257	31.777	38.993	43.746	47.296	35
31.355	16.855	32.944	40.426	45.354	49.034	36
33.121	17.408	34.025	41.752	46.842	50.643	37
34.935	17.915	35.017	42.969	48.207	52.120	38
36.798	18.376	35.919	44.076	49.449	53.462	39
38.710	18.792	36.731	45.072	50.567	54.670	40
40.000	19.042	37.220	45.672	51.239	55.398	40.662
40.669	19.162	37.455	45.961	51.564	55.748	41
42.677	19.490	38.096	46.747	52.445	56.701	42
44.734	19.777	38.656	47.434	53.216	57.535	43
46.839	20.025	39.141	48.029	53.884	58.257	44
48.992	20.238	39.557	48.540	54.457	58.876	45
51.194	20.418	39.910	48.973	54.943	59.402	46
53.444	20.570	40.207	49.337	55.352	59.844	47
55.742	20.696	40.454	49.640	55.691	60.211	48
58.089	20.800	40.656	49.889	55.970	60.513	49
60.000	20.869	40.791	50.054	56.156	60.713	49.799
62.927	20.952	40.954	50.254	56.380	60.956	51
65.419	21.006	41.060	50.384	56.526	61.113	52
67.960	21.049	41.143	50.486	56.640	61.236	53
70.548	21.082	41.207	50.565	56.729	61.332	54
73.185	21.107	41.257	50.626	56.797	61.406	55
75.871	21.126	41.294	50.672	56.848	61.462	56

78.605	21.141	41.322	50.706	56.887	61.504	57
80.000	21.147	41.334	50.720	56.903	61.521	57.57
81.387	21.151	41.343	50.732	56.916	61.535	58
84.218	21.159	41.358	50.751	56.937	61.558	59
87.097	21.165	41.369	50.764	56.952	61.574	60
100.000	21.175	41.390	50.789	56.980	61.604	
140.625	21.178	41.394	50.795	56.986	61.611	60
142.969	21.178	41.394	50.795	56.986	61.611	61
145.313	21.178	41.394	50.795	56.986	61.611	62
147.656	21.178	41.394	50.795	56.986	61.611	63
150.000	21.178	41.394	50.795	56.986	61.611	64
152.344	21.178	41.394	50.795	56.986	61.611	65
154.688	21.178	41.394	50.795	56.986	61.611	66
157.031	21.178	41.394	50.795	56.986	61.611	67
159.375	21.178	41.394	50.795	56.986	61.611	68
160.000	21.178	41.394	50.795	56.986	61.611	68.267
161.719	21.178	41.394	50.795	56.986	61.611	69
164.063	21.178	41.394	50.795	56.986	61.611	70
166.406	21.178	41.394	50.795	56.986	61.611	71
168.750	21.178	41.394	50.795	56.986	61.611	72
171.094	21.178	41.394	50.795	56.986	61.611	73
173.438	21.178	41.394	50.795	56.986	61.611	74
175.781	21.178	41.394	50.795	56.986	61.611	75
178.125	21.178	41.394	50.795	56.986	61.611	76
180.000	21.178	41.394	50.795	56.986	61.611	76.8
180.469	21.178	41.394	50.795	56.986	61.611	77
182.813	21.178	41.394	50.795	56.986	61.611	78
185.156	21.178	41.394	50.795	56.986	61.611	79
187.500	21.178	41.394	50.795	56.986	61.611	80
189.844	21.178	41.394	50.795	56.986	61.611	81

192.188	21.178	41.394	50.795	56.986	61.611	82
194.531	21.178	41.394	50.795	56.986	61.611	83
196.875	21.178	41.394	50.795	56.986	61.611	84
199.219	21.178	41.394	50.795	56.986	61.611	85
200.000	21.178	41.394	50.795	56.986	61.611	85.333
201.563	21.178	41.394	50.795	56.986	61.611	86
203.906	21.178	41.394	50.795	56.986	61.611	87
206.250	21.178	41.394	50.795	56.986	61.611	88
208.594	21.178	41.394	50.795	56.986	61.611	89
210.938	21.178	41.394	50.795	56.986	61.611	90
213.281	21.178	41.394	50.795	56.986	61.611	91
215.625	21.178	41.394	50.795	56.986	61.611	92
217.969	21.178	41.394	50.795	56.986	61.611	93
220.000	21.178	41.394	50.795	56.986	61.611	93.867
220.313	21.178	41.394	50.795	56.986	61.611	94
222.656	21.178	41.394	50.795	56.986	61.611	95
225.000	21.178	41.394	50.795	56.986	61.611	96
227.344	21.178	41.394	50.795	56.986	61.611	97
229.688	21.178	41.394	50.795	56.986	61.611	98
232.031	21.178	41.394	50.795	56.986	61.611	99
234.375	21.178	41.394	50.795	56.986	61.611	100
236.719	21.178	41.394	50.795	56.986	61.611	101
239.063	21.178	41.394	50.795	56.986	61.611	102
240.000	21.178	41.394	50.795	56.986	61.611	102.4
241.406	21.178	41.394	50.795	56.986	61.611	103
243.750	21.178	41.394	50.795	56.986	61.611	104
246.094	21.178	41.394	50.795	56.986	61.611	105
248.438	21.178	41.394	50.795	56.986	61.611	106
250.781	21.178	41.394	50.795	56.986	61.611	107
